# Fusion Patterns in the Skulls of Modern Archosaurs Reveal That Sutures Are Ambiguous Maturity Indicators for the Dinosauria

**DOI:** 10.1371/journal.pone.0147687

**Published:** 2016-02-10

**Authors:** Alida M. Bailleul, John B. Scannella, John R. Horner, David C. Evans

**Affiliations:** 1 Museum of the Rockies and Department of Earth Sciences, Montana State University, Bozeman, Montana, United States of America; 2 Royal Ontario Museum and Department of Ecology and Evolutionary Biology, University of Toronto, Toronto, Ontario, Canada; Medical University of South Carolina, UNITED STATES

## Abstract

The sutures of the skulls of vertebrates are generally open early in life and slowly close as maturity is attained. The assumption that all vertebrates follow this pattern of progressive sutural closure has been used to assess maturity in the fossil remains of non-avian dinosaurs. Here, we test this assumption in two members of the Extant Phylogenetic Bracket of the Dinosauria, the emu, *Dromaius novaehollandiae* and the American alligator, *Alligator mississippiensis*, by investigating the sequence and timing of sutural fusion in their skulls. As expected, almost all the sutures in the emu skull progressively close (i.e., they get narrower) and then obliterate during ontogeny. However, in the American alligator, only two sutures out of 36 obliterate completely and they do so during embryonic development. Surprisingly, as maturity progresses, many sutures of alligators become wider in large individuals compared to younger, smaller individuals. Histological and histomorphometric analyses on two sutures and one synchondrosis in an ontogenetic series of American alligator confirmed our morphological observations. This pattern of sutural widening might reflect feeding biomechanics and dietary changes through ontogeny. Our findings show that progressive sutural closure is not always observed in extant archosaurs, and therefore suggest that cranial sutural fusion is an ambiguous proxy for assessing maturity in non-avian dinosaurs.

## Introduction

The bones of the skulls of vertebrates are linked by different types of articulations, such as fibrous sutures in the cranial vault, the face and the palate, and cartilaginous synchondroses in the skull-base [1]. These articulations allow the growth of the skull during ontogeny (i.e., the life span of an organism). Numerous morphological studies on vertebrate skeletons, mostly focused on extant mammals and birds, have revealed a trend of progressive sutural closure (also referred to as sutural fusion) through ontogeny: sutures are generally open early in development and progressively close as maturity is attained [2–10]. It is also known that in some instances, this degree of sutural closure does not reflect ontogeny alone, but also reflects cranial biomechanics (e.g., relating to feeding behaviors [11]). In dinosaur paleontology, it has been assumed that sutural fusion is a good proxy for maturity in the Dinosauria (e.g., [12–19]). If a fossil dinosaur exhibits open sutures, it is often concluded that it is an *immature* specimen, while the presence of closed or obliterated sutures leads to the conclusion that it is skeletally *mature* (see the Methods section for our definitions of different morphological degrees of sutural fusion). In fact, the degree of sutural fusion is often the only criterion used to determine whether a fossilized individual represents a juvenile or adult, e.g., [12,18]. Moreover, the fusion of skull elements is sometimes used as a defining feature for taxa in cladistics analyses [17,20–22].

Accurately being able to determine ontogenetic stages in dinosaurs is critical to assessing their diversity and systematics [23–25]. Despite the pervasive use of sutural fusion to assess ontogeny in dinosaurs, it remains unknown if their juveniles did indeed consistently present open sutures and if more mature individuals showed closed (or obliterated) sutures. The first step in revisiting these assumptions is to test whether a correlation exists between fusion and maturity in the Extant Phylogenetic Bracket of Dinosauria (EPB [26]), with skeletonized specimens of known age or known relative maturity. Here, we present the results of a modified cladistic analysis (following the methodology of Brochu [27,28]) of skull growth series of two extant archosaurs, the emu (*Dromaius novaehollandiae)* and the American alligator (*Alligator mississippiensis)*, which are a living dinosaur and a member of their closest living relatives respectively. We used characters describing 1) the degree of closure and 2) the degree of interdigitation of some cranial, facial and palatal sutures, as well as some skull-base synchondroses. For the first time, we attempt to decipher the sequence and timing of cranial fusion in this group, and test whether the obliteration of any suture or any particular anatomical group of sutures coincide with the onset of sexual and/or skeletal maturity in these species. Histological analyses on ontogenetic series of emus and American alligator heads were also performed to complement our morphological observations with quantitative data. Our main question was: *Does the EPB of the Dinosauria follow a pattern of progressive cranial sutural closure through ontogeny*? Our results reveal undocumented complexity that has important implications for assessing relative maturity in non-avian dinosaurs.

## Materials and Methods

### Ethics Statement

The remains of American alligators were provided by Ruth Elsey, Rockefeller Wildlife Refuge, Grand Chenier, Louisiana (permit: AB070814 from the Louisiana Department of Wildlife and Fisheries) and emu heads were provided by Don Collins, from Montana Emu Ranch (MER; permit: 2354 from the Montana Department of Livestock). All specimens were cadaveric.

### Specimen Collection and Skeletonization

Almost all of the dry emu skulls (Table 1) were curated in the osteology collections of the Museum of the Rockies (MOR) and were collected from emu farms in 2013 and 2014 (MOR OST 1799 through 1813). Cadaveric fleshy heads were sent frozen to the MOR (from specimens that had died of natural causes) and defleshed by dermestid beetles (Skull Taxidermy, Deer Lodge, MT). A few emu specimens also came from the osteology collections of the Royal Ontario Museum (ROM). Nine out of the twenty-four skulls were aged by an emu rancher (D. Collins, MER) and only one of these specimens had a known sex (Table 1).

**Table 1:** List of the emu skulls used in this study.

Ontogenetic category	Specimen number	Skull length (mm)	Age	Sex	Geographic origin
**Juveniles**	ROM R7945	54.00 (e)	A few weeks (?)	?	?
	MOR OST 1799	54.99	A few weeks (?)	?	Montana
	ROM R7644	55.00 (e)	A few weeks (?)	?	?
	MOR OST 1805	55.82	A few weeks	?	Montana
	ROM R7630	61.00 (e)	A few weeks (?)	?	?
	MOR OST 1806	62.27	A few weeks	?	Montana
	MOR OST 1807	63.08	A few weeks	?	Montana
**Sub-adults**	MOR OST 1800	97.07	A few months (?)	?	Montana
	MOR-OST-1298	100.00 (e)	A few months (?)	?	Montana
	MOR OST 1808	116.67	8 to 10 months	?	Montana
	MOR OST 1802	128.36	A few months (?)	?	Montana
	MOR OST 1809	149.11	8 to 10 months	?	Montana
**Skeletally mature adults**	MOR OST 1810	143.94	18 months	?	California
	MOR OST 1814	151.9	18 months	?	California
	MOR OST 1815	153.13	18 months	?	California
	MOR OST 1811	154.39	18 months	?	California
	MOR OST 1813	154.54	18 months	?	California
	MOR OST 1297	155.00 (e)	> 10 months (?)	?	Montana
	MOR OST 186	155.00 (e)	> 10 months (?)	?	Montana
	MOR OST 1812	157.9	18 months	?	California
**Sexually mature adults**	MOR OST1803	152.23	20 years	M	Montana
	MOR OST 232	158.17	> 18 months (?)	?	Montana
	ROM R6843	163	> 18 months (?)	?	Australia
	ROM R7654	166	> 18 months (?)	?	?

The age is provided for 9 out the 24 specimens. The rest of the ages were estimated (those followed by a question mark) using their skull length. Abbreviations: (e), estimated; M, male.

The ROM provided almost all the American alligator skulls presented in this study; data available for the majority of these specimens included sex, total length, weight and geographic data (Table 2). Most of them were wild, but a few alligators were domestic. Only three specimens (hatchlings) had a known age. One fossil *Alligator* from the Pleistocene of Canada, with a skull length of 66 cm, was also examined (ROM R51011). Some alligator specimens from the osteology collections of the MOR were also included in this study (Table 2).

**Table 2:** List of the American alligator skulls used in this study.

Ontogenetic category	Specimen number	Skull length (mm)	Total length (cm)	Sex	W or D	Geographic origin	Preparation method
**Juveniles**	MOR OST 1646 (embryo)	28	102	?	?	Louisiana	Maceration
	ROM R7964	32.02	22.4	F	?	Florida	?
	MOR OST 1645 (embryo)	33	14.7	?	?	Louisiana	Maceration
	ROM R7966	34.7	28.3	M	?	Florida	?
	ROM R7965	38.83	24	M	?	Florida	?
	ROM R6251	42.99	31	?	?	South Carolina	?
	ROM R6252	43.1	29.8	?	?	South Carolina	?
	ROM R6253	43.33	19.8	?	?	South Carolina	?
	MOR OST 148	74	57.40 (e, M); 56.40 (e, F)	?	?	?	Dermestid beetles
	ROM R8352	105.64	80.5	M	W	Florida	Dermestid beetles
	ROM R8349	107.93	81.75	F	W	Florida	Dermestid beetles
	ROM R8350	111.03	86.5	M	W	Florida	Dermestid beetles
	MOR OST 820	119	90.50 (e, M); 89.90 (e, F)	?	?	?	?
	MOR OST 1028	121	91.90 (e, M); 91.30 (e, F)	?	?	?	Dermestid beetles
	ROM R8354	139.19	100	M	W	Florida	Dermestid beetles
	ROM R8355	147.97	113	M	W	Florida	Dermestid beetles
**Sub-adults**	ROM R8345	172.27	129	M	W	Florida	Dermestid beetles
	ROM R4405	175.82	138.43	M	D	Florida	Invert. Biota
	ROM R1698	195.74	?	?	?	Florida	?
	ROM R8335	202.55	161.3	M	W	Louisiana	Dermestid beetles
	ROM R4418	215.29	166.37	M	D	Florida	Invert. Biota
	ROM R8332	226.94	176.5	M	W	Louisiana	Dermestid beetles
	ROM R8334	244.72	198.1	F	W	Louisiana	Dermestid beetles
	ROM R8322	247.44	191.8	F	W	Florida	Maceration
	ROM R4420	247.93	182.88	M	D	Florida	Invert. Biota
	MOR OST 1029	250	184.30 (e, M); 186.00 (e, F)	?	?	?	Dermestid beetles
	ROM R8347	252.43	191.3	F	W	Florida	Dermestid beetles
**Sexually mature adults**	ROM R8323	254.86 (e)	204	F	W	Florida	Dermestid beetles
	ROM R4421	266.54	208.28	M	W	Florida	Invert. Biota
	ROM R8331	277.00 (e)	215.9	F	W	Louisiana	Dermestid beetles
	ROM R8344	288.52	203.2	M	W	Florida	Dermestid beetles
	ROM R8336	330	243.8	M	W	Louisiana	Dermestid beetles
	MOR OST 156	340	247.40 (e, M); 251.40 (e, F)	?	?	?	?
	ROM R8342	343	247	F	W	Florida	Dermestid beetles
	ROM R4422	346	236.22	F	W	Florida	Invert. Biota
	ROM R4416	350	242.97	F	D	Florida	Invert. Biota
	ROM R4401	357	269.88	F	D	Florida	Invert. Biota
	ROM R8343	370.00 (e)	264.1	F	W	Florida	Dermestid beetles or Invert.Biota
**Skeletally mature adults**	ROM R8326	373	274.3	F	D	Louisiana	Dermestid beetles
	ROM R8327	375 (e)	276.9	F	D	Louisiana	Dermestid beetles
	MOR OST 155	380	275.30 (e)	M	W	?	?
	ROM R4415	405	287.02	M	D	Florida	Invert. Biota
	ROM R8329	405	284.5	F	D	Louisiana	Dermestid beetles or Invert.Biota
	ROM R4411	445	330.2	M	D	Florida	Invert. Biota
	MOR OST 795	464	333.30 (e)	?	?	?	?
	ROM R8337	? (Braincase only)	360.7	M	W	Louisiana	Dermestid beetles or Invert.Biota
	ROM R8328	? (Braincase only)	375.9	M	D	Louisiana	Dermestid beetles or Invert.Biota
	ROM R8324	580	383.6	M	W	Florida	Dermestid beetles
	ROM R8333	? (Braincase only)	381	M	D	Louisiana	Dermestid beetles or Invert.Biota
**?**	ROM R51011	660	?	?	W	Canada (Pleistocene)	None

When total length was unknown (or if a specimen was incomplete), it was estimated using two equations [35,36]. The results from Female (F) and Male (M) equations are shown. Abbreviations: D, domestic; e, estimated; Invert. Biota, invertebrate biota; W, wild.

All the emu specimens were prepared by dermestid beetles, but the alligator specimens were skeletonized with various methods (Table 2). This information is important because the method of skull preparation (i.e., skeletonization) has an influence on the separation of adjoining but un-fused bones. Dermestid beetles are thought to be the best agents to conserve articulation. Maceration or boiling often leads to disarticulation (personal communications of various taxidermists) and may result in showing inaccurate sutural widths (i.e., sutures could appear wider, more ‘open’ than they actually are *in vivo* in heads with all associated soft-tissues).We aimed to eliminate this taphonomical bias by using mostly specimens skeletonized by dermestid beetles (or other invertebrates) and limited the use of macerated specimens (Table 2).

### Ontogenetic Categories

#### Dromaius novaehollandiae

Specimens of *D*. *novaehollandiae* were divided into four different ontogenetic categories (Table 1) using age, or skull length (SL, measured from the tip of the snout to the most caudal surface of the occipital condyle) and already-published growth patterns and ages at sexual and skeletal maturity on this species. It is safe to assume that emus older than 12 months are skeletally mature, and those older than 18 months are sexually mature and able to produce viable eggs [29–32]. The ontogenetic categories are as follows: 1) juveniles (from the time at hatching to a few weeks old, which corresponds in our sample to a SL between about 55 mm and 65 mm); 2) sub-adults (from a few months-old to 12 months-old, corresponding to a SL of 97 cm to about 150 cm); 3) skeletally mature individuals (from 13 to 18 months old, corresponding in our sample to skulls between 143 and 158 cm long) and 4) sexually mature individuals (older than 18 months old, which correspond in our sample to skulls between 152 cm to 166 cm). Note that these categories overlap due to intraspecific variation.

#### Alligator mississippiensis

We also distributed the specimens of *A*. *mississippiensis* into four different ontogenetic categories (Table 2). Since the ages of the specimens were unknown (except for three hatchlings), these categories were based on total length (TL) and the growth patterns already published for this species: Sub-adults have been designated by Woodward et al. [33], as individuals between 122 and 183 cm. The latter is the approximate length at which sexual maturity in attained, which is similar in populations from Louisiana [34] and Florida [33]. At a length of 270 cm, female alligators in Louisiana reach a significant plateau of growth [35] which corresponds to their skeletal maturity. Female alligators in Florida may keep growing to larger sizes (to about 300 cm, e.g., the longest female ever reported was 309.9 cm [36]. Males in Louisiana are skeletally mature at around 400 cm [35] but the longest alligator male ever reported was 426.9 cm in Florida [36].

When TL was unknown, it was estimated using SL and the equations of Chabreck and Joanen [35] and Woodward et al., [36]. The four ontogenetic categories are as follows: 1) juveniles (from the length at hatching through 121cm); 2) sub-adults (from 122 to 200 cm), 3) sexually mature (from 201 to 270 cm) and 4) skeletally mature individuals (larger than 271 cm). Three matters are important to mention: 1) a full term embryo (MOR OST 1645) was treated as a juvenile; 2) since Woodward et al., [33] found that only 64% of females larger than 183 cm long are sexually mature, we assumed a TL of 200 cm for sexual maturity; and 3) it is safe to assume that all the females larger than 270 cm are skeletally mature since they all come from a population in Louisiana. However, it is not guaranteed that all males larger than 270 cm are skeletally mature, instead, they might be *circum* skeletal maturity. We note that it has previously been assumed that American alligators have indeterminate growth, but by means of histological analysis, Woodward and colleagues recently showed that they eventually stop growing (at least, their bone circumference does) and present determinate growth [37].

### Modified Cladistics Methodology

This modified cladistic methodology was developed by Brochu [27,28] and it has seen an increase in use within the last few years, especially among paleobiologists interested in maturity assessment [14,38–40]. In this method, immature character states are analogous to the plesiomorphic conditions of phylogenetic analyses, while mature character states are analogous to the apomorphic conditions. Shared traits represent synontomorphies, following the terminology of Frederickson and Tumarkin-Deratzian [40]. It is a powerful method because unlike classic cladistics which generate a multi-taxa cladogram, cladistics ontogeny can be used to produce a single-species ontogram, e.g., see [14,38–40]. With this ontogenetic pattern, it is possible to identify ontogenetic hierarchies, such as a hierarchy of sutural obliteration in the present study. By using extant specimens of known age and/or relative ontogenetic category, this method can help test if progressive sutural closure is indeed a good proxy of maturity. If the phylogenetic analysis produces an ontogram that is consistent with ontogeny (i.e., if the least mature individuals are near the base of the tree and the progressively more mature individuals get further away from the root), this would indicate that progressive sutural closure does occur during ontogeny and that it is a reliable indicator of maturity in this species. Secondly, the timing of sexual and skeletal maturity can be mapped onto this hierarchy.

**Fig 1:**
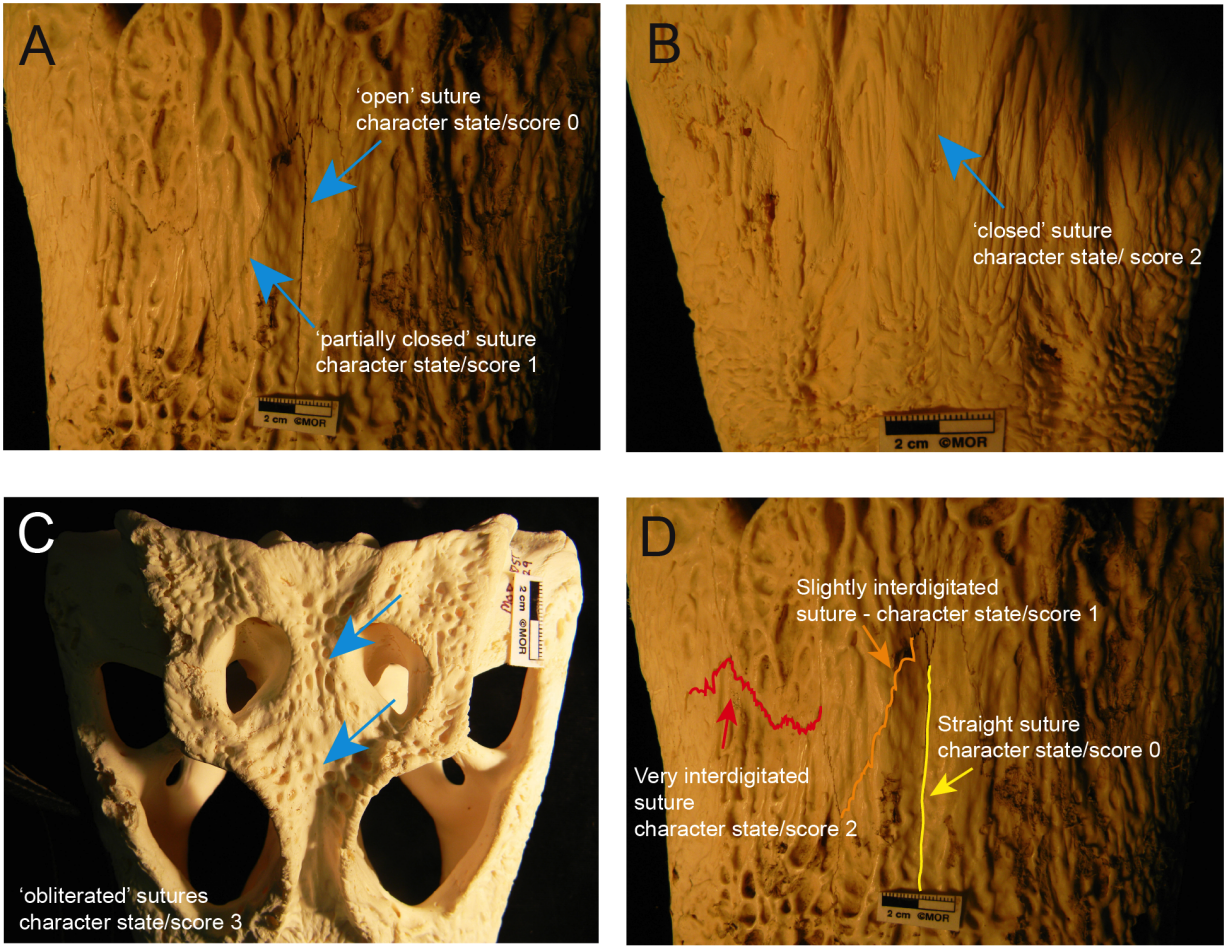
Character states and scores describing the degree of sutural closure and interdigitation in some American alligator skulls. **A**, The blue arrows show an open and a partially closed suture on MOR OST 155. **B**, The blue arrow shows a closed suture (with its suture line still visible) on MOR OST 1029. **C**, The blue arrows show two obliterated sutures (with their suture lines morphologically invisible) on MOR OST 1029. **D**, The colored lines show the different degrees of interdigitation: a straight (yellow line), slightly interdigitated (orange line) and a very interdigitated suture (red line) on MOR OST 155. These interdigitation degrees are based on those in Herring [82].

Two analyses were employed on 24 dry emus skulls (Table 1) and 49 dry alligator skulls (Table 2) in order to explore the hierarchy of obliteration for their skull sutures and skull-base synchondroses (Table 3). Immature character states were coded as zeroes and mature character states were coded as ones or higher numbers (Fig 1). A hypothetical embryo where all character states were immature (all-zero), was designated as the outgroup to polarize the characters. A total of 20 and 37 sutures were examined in the emu and American alligator respectively, and each suture was coded according to its degree of sutural closure (Fig 1A–1C) and its degree of interdigitation (Fig 1D). We included sutural interdigitation in our coding because this parameter often increases during ontogeny in extant species [5] and it has previously been used to estimate maturity in some fossil dinosaurs, e.g., [41]. Before going further into the description of our coding, it is important to note that for the purposes of the present study, we consider the terms ‘sutural closure’ and ‘sutural fusion’ synonymous (only at the morphological level), because they have often been employed interchangeably in the dinosaur paleontology literature (e.g., [14]). However, as previous authors have already pointed out [3,5,28,42], we are aware that the true degree of fusion of a suture (i.e., when sutural bony margins come into contact) can only be assessed histologically (e.g., with the obliteration index, [43]).

**Table 3:** List of sutures and synchondroses morphologically examined in this study.

Anatomical Group	Sutures/Synchondroses	*D*. *novaehollandiae*	*A*. *mississippiensis*
**Facial sutures**	nasal-mesethmoid	x	
	nasal-prefrontal	x	x
	nasal-frontal	x	x
	nasal-premaxilla	x	x
	frontal-mesethmoid	x	
	internasal		x
	nasal-maxilla		x
	maxilla-jugal		x
	maxilla-premaxilla		x
	maxilla-lachrymal		x
	prefrontal-lachrymal		x
	lachrymal-jugal		x
	frontal-prefrontal		x
	interpremaxillary		x
**Cranial sutures**	interfrontal	x	x
	frontal-parietal	x	x
	interparietal suture	x	x
	parietal-squamosal	x	x
	supraoccipital-parietal	x	x
	exoccipital-squamosal	x	x
	laterosphenoid-parietal	x	
	laterosphenoid-squamosal	x	
	frontal-postorbital		x
	postorbital-squamosal		x
**Palatal sutures**	pterygoid-vomer	x	
	vomer-palatine	x	
	maxilla-premaxilla	x	x
	premaxilla-vomer	x	
	intermaxillary		x
	maxilla-palatine		x
	interpalatine		x
**Braincase synchondroses (or articulations)**	exoccipital-supraoccipital	x	x
	exoccipital-basioccipital	x	x
	laterosphenoid-prootic		x
	laterosphenoid-basisphenoid		x
	laterosphenoid-pterygoid		x
	laterosphenoid-quadrate		x
	basisphenoid-prootic		x
	basisphenoid-basioccipital	x	x
	basisphenoid-pterygoid		x
	exoccipital-prootic		x
	exoccipital-basioccipital		x
	exoccipital-quadrate		x
	quadrate-pterygoid		x

The degree of sutural closure (or sutural fusion) refers to the width of the gap seen externally between adjacent bones. Scoring for these characters is a modified version of Ryan et al., [44] and Frederickson and Tumarkin-Deratzian [40] and is as follows: sutures were considered open (coded as a zero) if bones were clearly separate along their margin of contact; partially closed (coded as a one) if bones were still separate along their margin of contact, but were significantly closer to each other than in the previous state; closed (coded as a two) if the bones were conjoined into a single unit, with a surface nearly level with the surrounding bone, but whose sutural line was still visible externally and could still be traced; and finally, completely obliterated (coded as a three) when there was absolutely no trace of the suture line on the surface of the bones (Fig 1A–1C).

For the degree of interdigitation, sutures were coded based on their morphology and sinuosity: straight (coded as a zero), slightly interdigitated (coded as a one), and very interdigitated (coded as a two; Fig 1D). For four sutures (premaxilla-maxilla, parietal-supraoccipital, frontoparietal, basisphenoid-basioccipital), contact points with surrounding bones were visible in multiple views (e.g., dorsal, palatal, ectocranial or endocranial views) allowing the assessment of the potential progression of fusion throughout the suture. This gave a total of 42 characters for the emus and 80 characters for the alligators. All specimens and characters were compiled into two data matrices (Text A, Dataset A, Dataset B in S1 File) using Mesquite [45]. Polymorphic characters were coded by scoring all states in the same cell of the character matrix (e.g., ‘0 & 1 & 2’ if all three states were present in one same suture). The cladistic analyses were implemented with Phylogenetic Analysis Using Parsimony* v. 4.0b10 (PAUP*[46]). A heuristic search was used with ACCTRAN character optimization methods. This heuristic search implemented the random addition sequence with tree-bisection-reconnection (TBR) branch swapping and 1000 replicates. All characters were equally weighted and left unordered. Maxtrees was set up to 5000 for the alligator analysis. The emu analysis exceeded 5000 trees and thus 10000 Maxtrees were set in this case. Support for ontogenetic grouping was determined using non-parametric bootstrap resampling in PAUP* [47]. Five thousand bootstrap replicates were analyzed with one tree per replicate retained. Trees were then visualized with FigTree v. 1.4.0 (http://tree.bio.ed.ac.uk/software/figtree/).

To assess the repeatability of our sutural closure and sutural interdigitation character codings, a total of eight specimens with 42 characters each (giving a total of 336 character states/measurements) were coded twice by the first author at a one-year interval. For the two character matrices, we estimated inter-rater reliability between the two observation points using the R package "irr" [48]. Inter-rater reliability was highly significant (Cohen's κ = 0.62; *Z* = 19.1; P << 0.00001; number of errors = 90). Similarly, the Pearson’s product-moment correlation between two sets of characters was highly significant (ρ = 0.649, P << 0.00001).

### Averaged Degrees of Sutural Closure and Interdigitation

After the cladistic analyses were performed, we re-used the phylogenetic matrices for another purpose: we treated the character state(s) found in each cell as a ‘score’, in order to calculate averages of sutural closure (between zero and three) and interdigitation (between zero and two) for each specimen, and observe how these averages change through ontogeny. This scoring (the same as that presented in Fig 1) is a modified version from that found in Wilson and Sánchez-Villagra [49] concerning the sutures of hystricognath rodents (see their Fig 4). In the present study, since the scores came from matrices previously used for cladistics, if a suture presented multiple states at once (e.g., open (0) in one area, and partially closed (1) in another) the score of the suture became an average of each character state (e.g., score (0.5) for this example). In the case of Wilson and Sanchez Villagra [49], such sutures had specific set scores (see their Fig 4B and 4C for sutures that are ¼ or ½ closed; also see [42]). We calculated these averaged degrees of sutural closure and interdigitation in each specimen and in each of the four ontogenetic categories for the two species (Tables A–D in S1 File).

### Histological and Histomorphometric Analyses

As mentioned previously, we are aware that the coding that we describe at the morphological level might not necessarily reflect the actual histological state of the sutures (e.g., a suture that appears ‘open’ morphologically could actually be partly obliterated/fused internally, or *vice versa*; see [3,5,28,42]). Therefore, in order to complement our morphological analyses, we histologically sampled a cranial and a facial suture (the frontoparietal and the internasal sutures respectively) and a skull-base synchondrosis (basioccipital-exoccipital) in two ontogenetic series of emu and American alligator heads and skulls (Table 4). A total of twelve specimens were sectioned (six per growth series), but only six of them are presented here (Table 4) since they will be fully described in another study [50].

**Table 4:** List of the specimens sectioned in the present study.

Species	Specimen number	Skull length (cm)	Provenance/D or W	Estimated age	Ontogenetic category	Skull/Head & embedding media
*Dromaius novaehollandiae*	MOR OST 1799	5.5	Montana/D	a few weeks	juvenile (hatchling)	dry skull/Epoxy
	MOR OST 1801	~12.8	Montana/D	8 to 10 months*	juvenile	head/PMMA
	MOR OST 1803	15.2	Montana/D	20 years (male)*	skeletally mature	dry skull/PMMA
*Alligator mississippiensis*	MOR OST 1647	2.5	Louisiana/?	a few days	juvenile (hatchling)	head/PMMA
	MOR OST 1797	15.5	Louisiana/?	4–5 years	sub-adult	head/PMMA
	MOR OST 1798	28.5	Louisiana/W	9–12 years	sexually mature	head/PMMA

The same articulations were systematically chosen and sectioned in each specimen: 1) the fronto-parietal suture (cranial), 2) the internasal suture (facial) and 3) the basioccipital-exoccipital synchondrosis (skull-base). Ages and ontogenetic categories were estimated and based on skull length and on the literature available on the growth trajectories of these species (for the emu see [30,31] and for the American alligators see [35,36]). The asterisks indicate that the age was known. Abbreviations: D, domestic; PMMA, Poly-methyl-methacrylate; W, wild.

Fragments possessing the sutures/synchondroses of interest were extracted from frozen heads or dry skulls using a dremel and a diamond blade. These fragments were then fixed in 10% neutral buffered formalin solution for three to four days (with one solution change per day), transferred to a solution of 70% Ethanol (EtOH) and serially dehydrated in graded solutions of 80%, 95% and finally 100% EtOH. Specimens were then cleared in xylene and embedded in Poly-methyl-methacrylate (PMMA) or Epoxy resin (Table 4). They were then treated according to the techniques established by Lamm for small fossil thin-sectioning [51]. Thick sections (between 1.0 and 1.3 mm) were cut for each suture/synchondrosis with a Norton 5” or 7” diamond-edge blade on an Isomet precision saw (Buehler). These sections were mounted on plexiglas slides with cyanoacrylate glue and ground by hand on a Buehler Ecomet grinder with water and silicon carbide paper of decreasing grit sizes: 320, 400, 600 and 800. Finally, they were polished by hand with wet polishing cloths and aluminum oxide powder (5μm then 1μm), giving a final thickness of about 100μm. They were stained with Toluidine-Blue (wich stains bone dark blue and cartilage purple) and studied by light microscopy under normal light with a Nikon Optiphot-Pol polarizing microscope. Photographs were taken with a Nikon DS-Fi1 digital sight camera and the NIS Elements BR 4.13 software.

**Fig 2:**
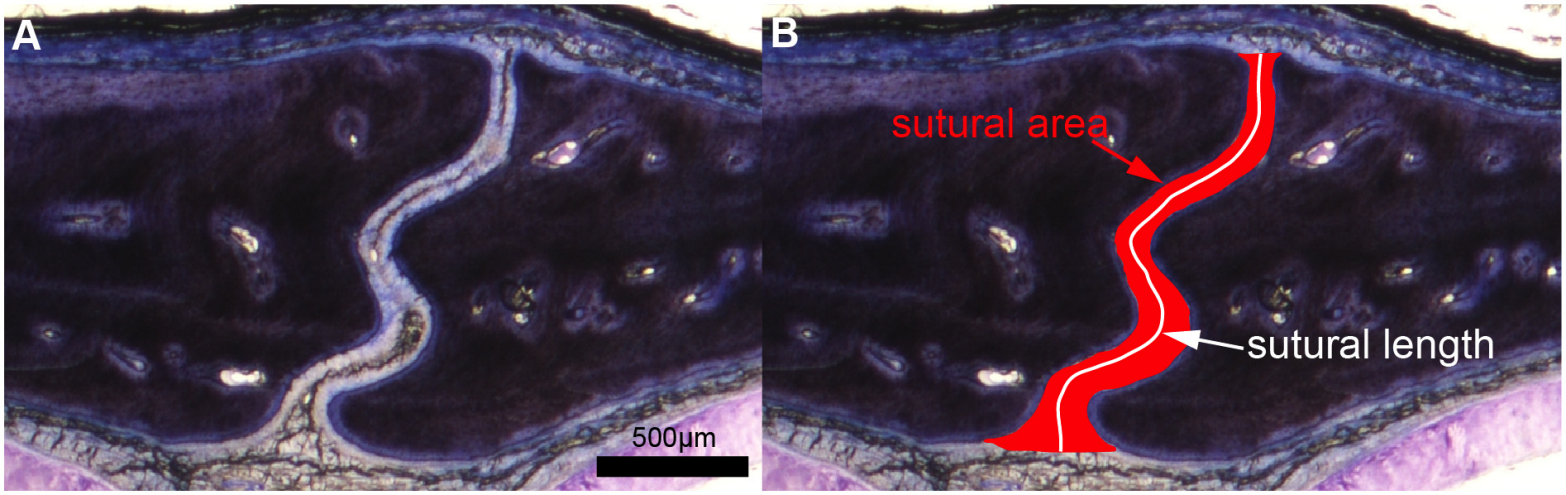
Photomicrographs of a transverse section through an alligator internasal suture showing measurements of sutural area and sutural length. **A,** An alligator internasal suture. **B**, Measurements of sutural area and sutural length. The ratio between sutural area (in red) and sutural length (in white) gives the averaged width of the suture. The sections are stained with Toluidine-blue.

The average sutural width (and synchondroseal width) was measured from nine photomicrographs of American alligator heads (with their associated soft-tissues; MOR OST 1647, 1797, 1798). Heads, but not skulls, were used to rule out potential taphonomical biases. Previous authors have calculated and measured sutural widths by various methods (e.g., measuring the width of the suture at ten different intervals and then calculating a mean value [52]; also see methods in [53–55]). Here, we present another way to quantify sutural width: we used the ratio between the sutural area in μm^2^ and the total length of the suture in μm (see the example in Fig 2). The length of the suture represents the length of the central suture line starting from the ectocranial side and ending on the endocranial side. For the synchondrosis, the area measured corresponds to the area occupied by hyaline cartilage (unmineralized cartilage). Areas and lengths were manually drawn and measured using scaled photomicrographs in Adobe Illustrator CS6 and the AreaLength plug-in.

## Results

### Ontograms: sequences and timings of sutural closure

Growth series of the skulls of the emu (*Dromaius novaehollandiae*, n = 24; Table 1*)* and the American alligator (*Alligator mississippiensis* n = 50; Table 2*)*, were analyzed in order to determine the hierarchy of sutural closure. As mentioned earlier, the ontogenetic characters coded in our analysis describe different degrees of closure (Fig 1) for cranial, facial and palatal sutures, as well as skull-base synchondroses (Table 3).

#### Dromaius novaehollandiae

**Fig 3:**
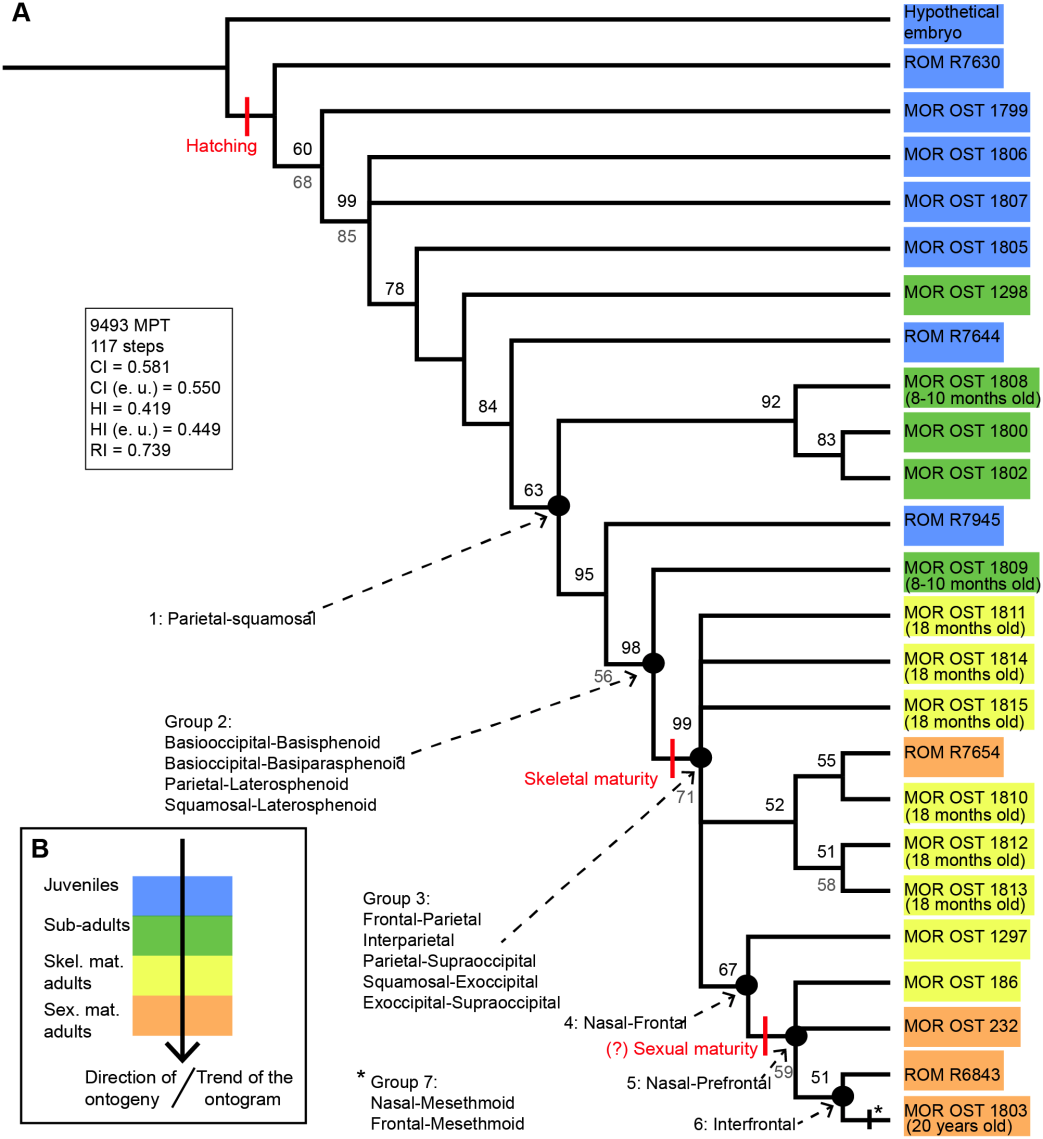
Ontogram of *D*. *novaehollandiae* (n = 24). **A**, This ontogram is the 50% majority rule consensus tree for 9493 MPT. The onset of hatching, skeletal maturity and (approximate) sexual maturity are mapped on this diagram. A precise sequence of sutural obliteration is given by synontomorphies on the left of the ontogram (i.e., the sutures or group of sutures that obliterate consistently in all the specimens of the analysis).Bootstrap support values are in grey below nodes, and the percent occurrence for nodes are in black above horizontal lines.**B,** Color code indicating the ontogenetic category of each specimen. Categories were estimated prior to phylogenetic analysis in each specimen and were based on known age or on skull length (Table 1) and already-published growth patterns on this species. This color code is the same in A. Note that the ‘trend’ of the ontogram follows the direction of ontogeny in general. **Abbreviations**: **CI**, consistency index; **e.u.**, excluding uninformative characters; **HI**, homoplasy index; **MPT**, most parcimonious trees; **RI**, retention index; **Skel. mat**., skeletally mature; **Sex. mat.**, sexually mature.

The cladistic analysis of 24 specimens for 42 multistate characters gave a strict consensus that collapsed into the hypothetical embryo and one large polytomy for all the other specimens. Therefore, in order to provide some structured data we show the 50% majority-rule consensus tree (Fig 3A). The tree is linear and pectinate, with three groups collapsing into polytomies. It has a length of 117 steps, a consistency index of 0.58, a homoplasy index of 0.42 and a retention index of 0.74. The color code (Fig 3B) shows the ontogenetic categories that were estimated prior to phylogenetic analysis in each specimen (see Methods section). The trend of this ontogram is consistent with progressive fusion throughout ontogeny: the least mature individuals are near the base of the tree (in blue and green) and the progressively more mature individuals are further away from the root (in yellow and orange). Only three groups of specimens seem to deviate from this ‘correct’ pattern: MOR OST 1298 or ROM R7644, ROM R7945 or MOR OST 1809, and ROM R7654. Aside from these inconsistencies, the ontogram reproduced a pattern consistent with ontogeny; the sutures of emus are mostly open in juveniles, then progressively close and obliterate during ontogeny (with 75% of obliteration in a 20 year-old male emu). This pattern of progressive sutural closure is expected and does not contradict the assumption mentioned in the introduction. Fig 3A shows selected synontomorphies concerning sutural obliteration, i.e., the sutures that obliterate consistently in all of the specimens of the analysis (coded as a three as in Fig 1C). The sequence of obliteration of the sutures and synchondroses in *D*. *novaehollandiae* is described as follows (Fig 3A): (1) the parieto-squamosal suture, (2) the basioccipital-basisphenoid synchondrosis and the parietal-laterosphenoid and the squamosal-laterosphenoid sutures, (3) the fronto-parietal, interparietal, parietal-supraoccipital and the squamosal-exoccipital sutures, and the exoccipital-supraoccipital synchondrosis, (4) the naso-frontal suture, (5) the naso-prefrontal suture, (6) the interfrontal suture, and (7) the nasal-mesethmoid and frontal-mesethmoid sutures. These last two obliterations occur in the specimen MOR OST 1803, which is the furthest away from the root and the oldest of our sample (a 20 year-old male). The onset of skeletal maturity coincides with the obliteration of the sutures of group (3) listed above, while the onset of sexual maturity corresponds to the obliteration of the nasal-prefrontal and the naso-frontal sutures. To roughly summarize this sequence, the braincase synchondroses and the cranial sutures are the first parts of the skull to obliterate completely, while facial sutures obliterate last. No palatal suture obliterated in a consistent pattern within all the specimens examined.

#### Alligator mississippiensis

**Fig 4:**
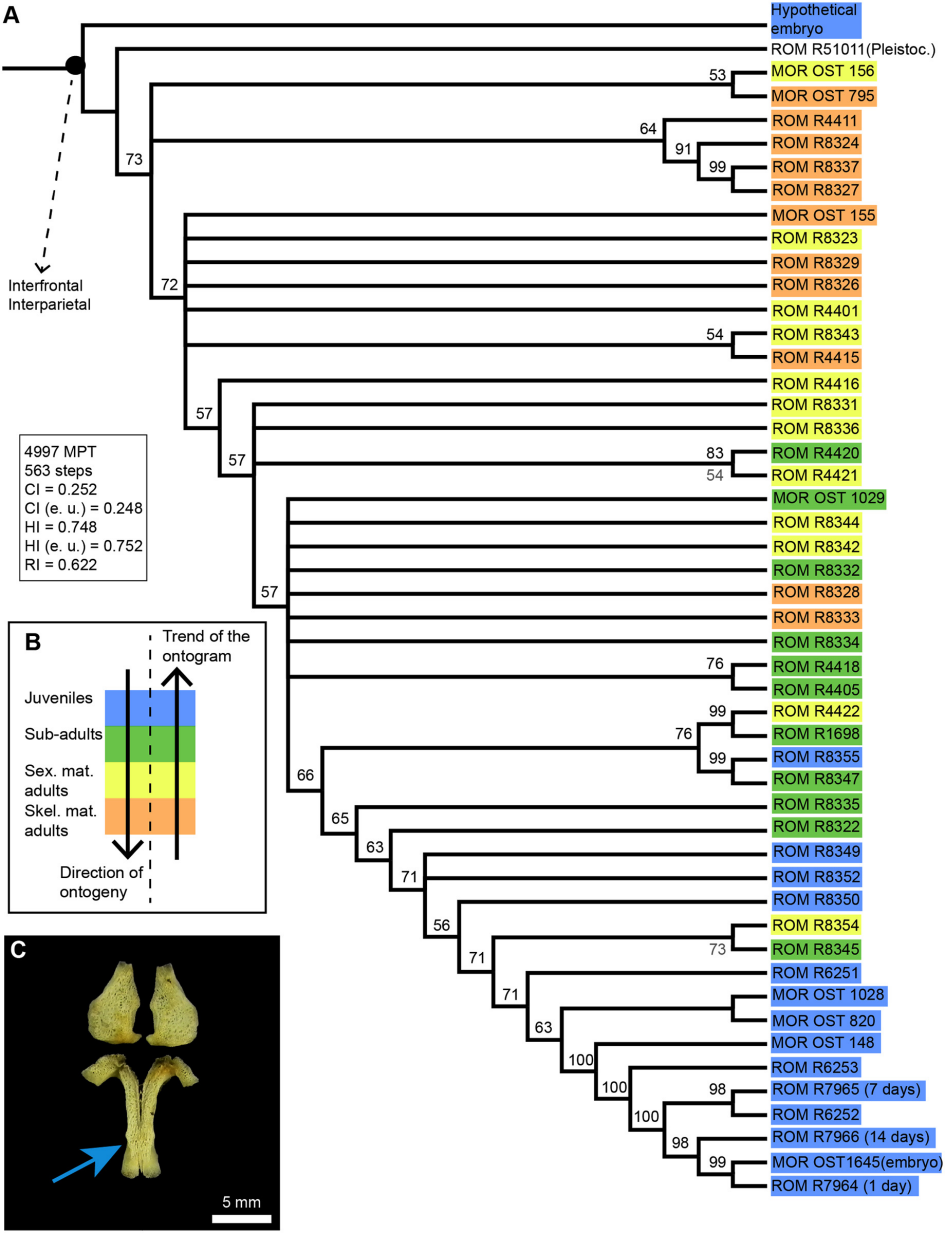
Ontogram of *A*. *mississippiensis* (n = 49). **A**, This ontogram is the 50% majority rule consensus tree for 4997 MPT. The analysis only produced two synontomorphies concerning sutural obliterations (the interfrontal and interparietal sutures). Bootstrap support values are in grey below nodes, and the percent occurrence for nodes are in black above horizontal lines. **B**, Color code indicating the ontogenetic category of each specimen in A. These categories were estimated prior to phylogenetic analysis in each specimen, based on known skull length, total length, sex and geographic provenance (Table 2), as well as previously published growth patterns for this species. Note that in general, the ‘trend’ of the ontogram is opposite to the correct direction of ontogeny. **C**, Paired frontals (bottom) and parietals (top) of an unhatched embryo (MOR OST 1646). The blue arrow indicates an anterior-posterior gradient of fusion. **Abbreviations**: **CI**, consistency index; **e.u**., excluding uninformative characters; **HI**, homoplasy index; **MPT**, most parcimonious trees; **Pleistoc.**, Pleistocene; **RI**, retention index; **Skel. mat.**, skeletally mature; **Sex. mat**., sexually mature.

The cladistic analysis of 49 specimens for 80 multistate characters gave a strict consensus that collapsed into the hypothetical embryo and multiple polytomies (S1 Fig). Therefore, we document the 50% majority rule consensus tree (Fig 4A). The tree is linear and pectinate, with four groups collapsing into polytomies. It has a length of 563 steps, a consistency index of 0.25, a homoplasy index of 0.75 and a retention index of 0.62. The pattern produced by the alligator ontogram is opposite to that of the emu: the most mature individuals are near the base of the tree (in yellow and orange) and the less mature individuals are further away from the root (in blue and green). For example, the specimen that falls right next to the root is the enormous fossil *Alligator* skull (66 cm long) from the Pleistocene, while the animals furthest away from the root are an embryo (MOR OST 1645) and a one day-old hatchling (ROM R 7964). This pattern can the explained by the occurrence of sutures which progressively become wider, rather than become narrower and fuse during the ontogeny of the American alligator. Sutures are more open (i.e., wider) in the most mature individuals compared to the least mature individuals Moreover, only two sutures obliterate completely during the ontogeny of *A*. *mississippiensis*: the interfrontal, followed by the interparietal suture (Fig 4A and 4C; which gives only 5% of obliteration). Obliteration of these two sutures occurs during embryonic development (see Fig 4C that shows the fusion of the frontal bones in an unhatched embryo, MOR OST 1646, not included in this cladistic analysis). The full-term un-hatched embryo (MOR OST 1645) had already obliterated these two sutures. Thus, while the emu presents the expected pattern of progressive sutural closure (with sutures becoming narrower and then obliterating), the American alligator does not and, in fact, its sutures appear to be widening.

### Averaged degrees of sutural closure

**Fig 5:**
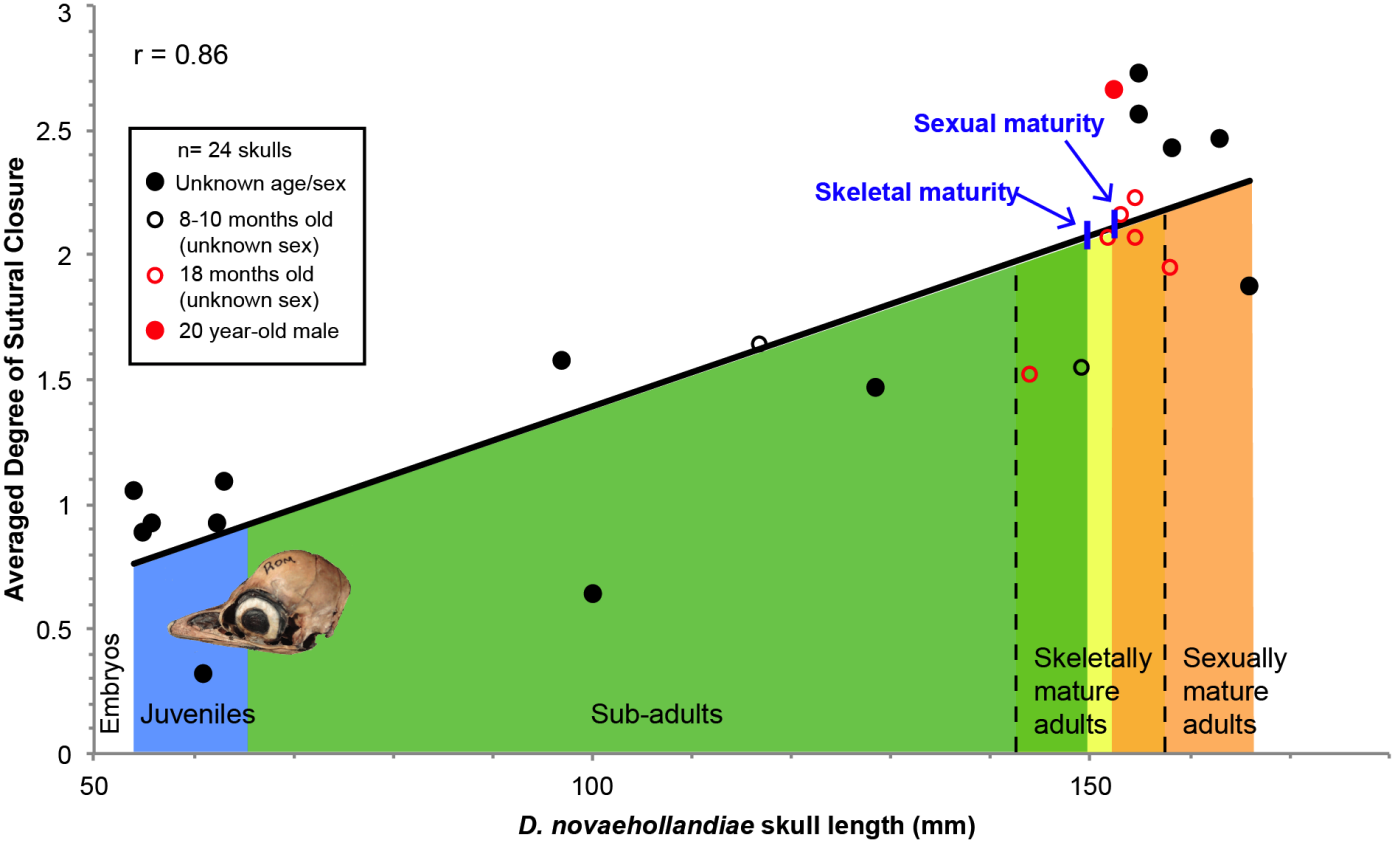
Linear relationship between ontogeny (using skull length as a proxy) and the averaged degree of sutural closure in *D*. *novaehollandiae* (n = 24). If the previously mentioned character states of each suture are transformed into set scores and if averages of closure are calculated for each specimen consequently, this type of data visualization can be generated. An average of zero for one specimen would mean that all its sutures are open, while an average of three would mean that all its sutures are obliterated. This relationship has a Pearson’s coefficient of correlation (r) of 0.86. The approximate onsets of skeletal and sexual maturity are mapped on the trend line. Below the trend line are the different ontogenetic categories, indicated with the same color code as that in Fig 3B. Each data point (i.e., each skull) is characterized by its age (see legend in black square). This relationship shows an obvious increase in the degree of sutural closure as ontogeny proceeds, in other words sutures become more closed.

**Fig 6:**
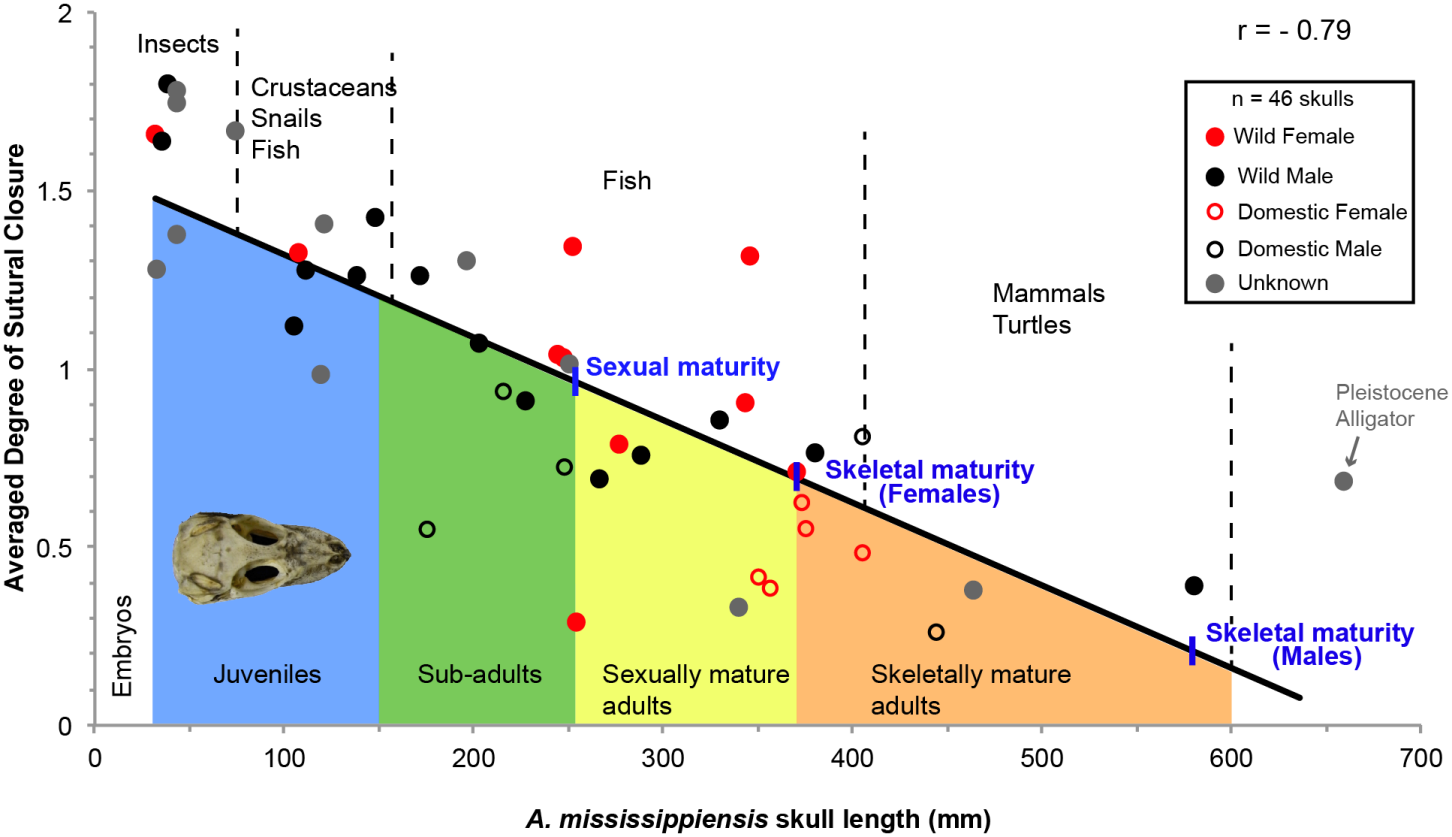
Linear relationship between ontogeny (using skull length as a proxy) and the averaged degree of sutural closure in *A*. *mississippiensis* (n = 46). This relationship has a Pearson’s coefficient of correlation (r) of -0.79. The approximate onsets of sexual maturity and skeletal maturity for females and males are mapped on the trend line. Below the trend line are the different ontogenetic categories, indicated with the same color code as that used in previous figures. Above the trend line are exposed the estimated diet of these animals at the time of death, based on previous studies [71,72]. Each data point (i.e., each skull) is characterized by its sex and domestic or wild status (see legend in black square). This relationship shows an obvious decrease in the degree of sutural closure as ontogeny proceeds, in other words, sutures become wider.

As described in the Methods sections, we ‘transformed’ the character states of each suture into a set score (Fig 1) in order to calculate the global degree of sutural closure for each individual specimen and also for each ontogenetic category (Figs 5 and 6). An average of zero in one specimen would mean that all its sutures are open, while an average of three would mean that all its sutures are obliterated. Averages were also calculated for each anatomical group of sutures (S2 Fig). The global averaged degree of interdigitation was also calculated for each ontogenetic category (S3 Fig). Assigning scores to sutures provides a separate type of visualization of the data compared to that provided by the ontograms.

The relationship between the averaged degree of sutural closure per individual and ontogeny is shown using skull length as a proxy in 24 emus (Fig 5) and 46 American alligators (Fig 6). Three alligators were excluded from this data set because of their incompleteness (only their braincases remained). Note that the categories show moderate overlap in Fig 5. This discrepancy is likely due to intraspecific variation. The emu dataset shows a clear linear relationship between sutural closure and maturity, with a Pearson’s correlation coefficient of 0.86. Results are consistent with the ontogram, the degree of sutural closure increases during ontogeny, with the lowest average (0.32) present in the juvenile ROM R7360 (estimated to be a few weeks old) and the highest average (2.73) present in what we estimated to be a skeletally mature adult (of unknown age, MOR OST 186). The second highest average (2.67) was that of MOR OST 1803, the twenty year-old male (see Table A in S1 File for the averages of all the specimens). The averaged degrees of sutural closure were also calculated for each ontogenetic category: the juveniles altogether show a value of 0.91, the sub-adults show 1.45, the skeletally mature individuals show 2.10, and the sexually mature individuals show 2.52 (see Table A in S1 File).

The American alligator dataset exhibits a clear inverse relationship between the degree of sutural closure and skull length, with a Pearson’s correlation coefficient of -0.79 for a linear relationship. However, note that this relationship could potentially be logarithmic instead, since it shows a slightly better Pearson’s coefficient of -0.82. This data supports what was documented by the ontogram (Fig 4A). The highest averaged degree of sutural closure (1.8) is present in ROM R6252, a hatchling, while the lowest (0.26) is presented by a skeletally mature domestic male ROM R4411 (see Table B in S1 File for the averages of all the specimens). The averaged degrees of sutural closure were also calculated for each ontogenetic category: the juveniles altogether have the highest value of 1.45, the sub-adults have 1.02, the sexually mature individuals have 0.68, and the skeletally mature individuals have the lowest value of 0.53 (see Table B in S1 File). Note that these averages are smaller than what was observed in the ontogenetic categories of the emu listed previously. These data show that while the skulls of emus do progressively fuse (Fig 5), the skull sutures of alligators appear to get wider during ontogeny (Fig 6). However, even though the two trends shown by alligators and emus are opposite, it would appear that they still show a conserved pattern of sutural fusion: in the emu, all the skull-base synchondroses are obliterated first, which are then followed by cranial sutures and finally facial sutures. Palatal sutures do not obliterate (S2 Fig). This pattern differs from the sequence published for mammalian skulls [6,7]. In *A*. *mississippiensis*, at any given time during ontogeny, cranial sutures present the highest degree of closure, followed by skull-base synchondroses and facial sutures. Palatal sutures are the most opened of all (S2 Fig). This hierarchy is rather similar to that presented in the emu and may suggest some conservation of sutural pattern across these two archosaurian species.

In the following section, we use histological analyses to verify that the two opposing trends (Figs 5 vs. 6) are also observed microscopically.

### Histology and Histomorphometry

**Fig 7:**
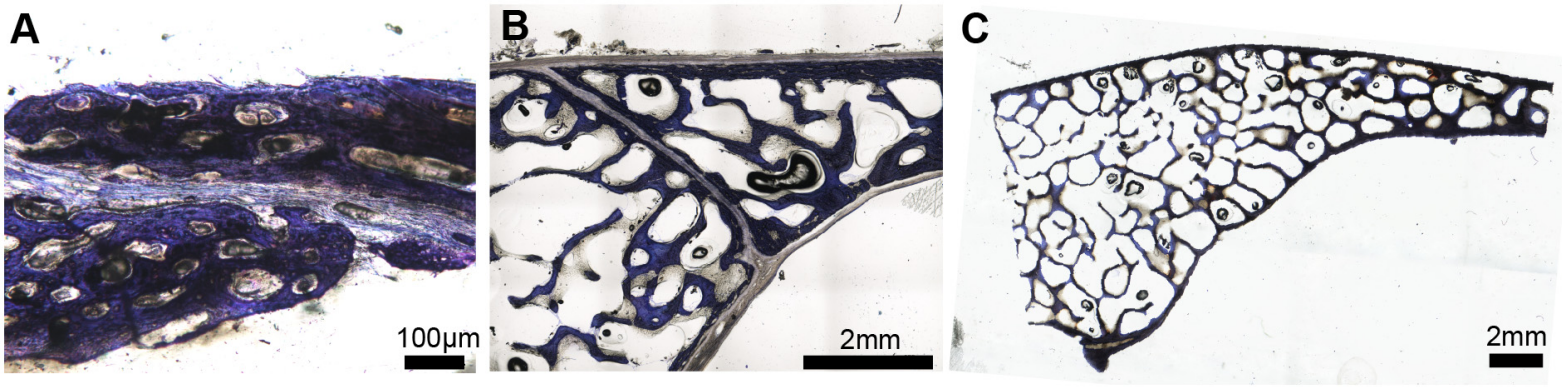
Parasagittal sections through the frontoparietal suture in an ontogenetic series of emus, stained with Toluidine-blue. **A,** Open suture in MOR OST 1799, a specimen a few weeks old. **B,** Open suture in MOR OST 1801, an 8 to 10 month old specimen. **C**, Obliterated suture in MOR OST 1803, a 20 year-old male. This suture is replaced by trabeculae of lamellar bone. The average sutural widths are marked in red on each photomicrograph.

**Fig 8:**
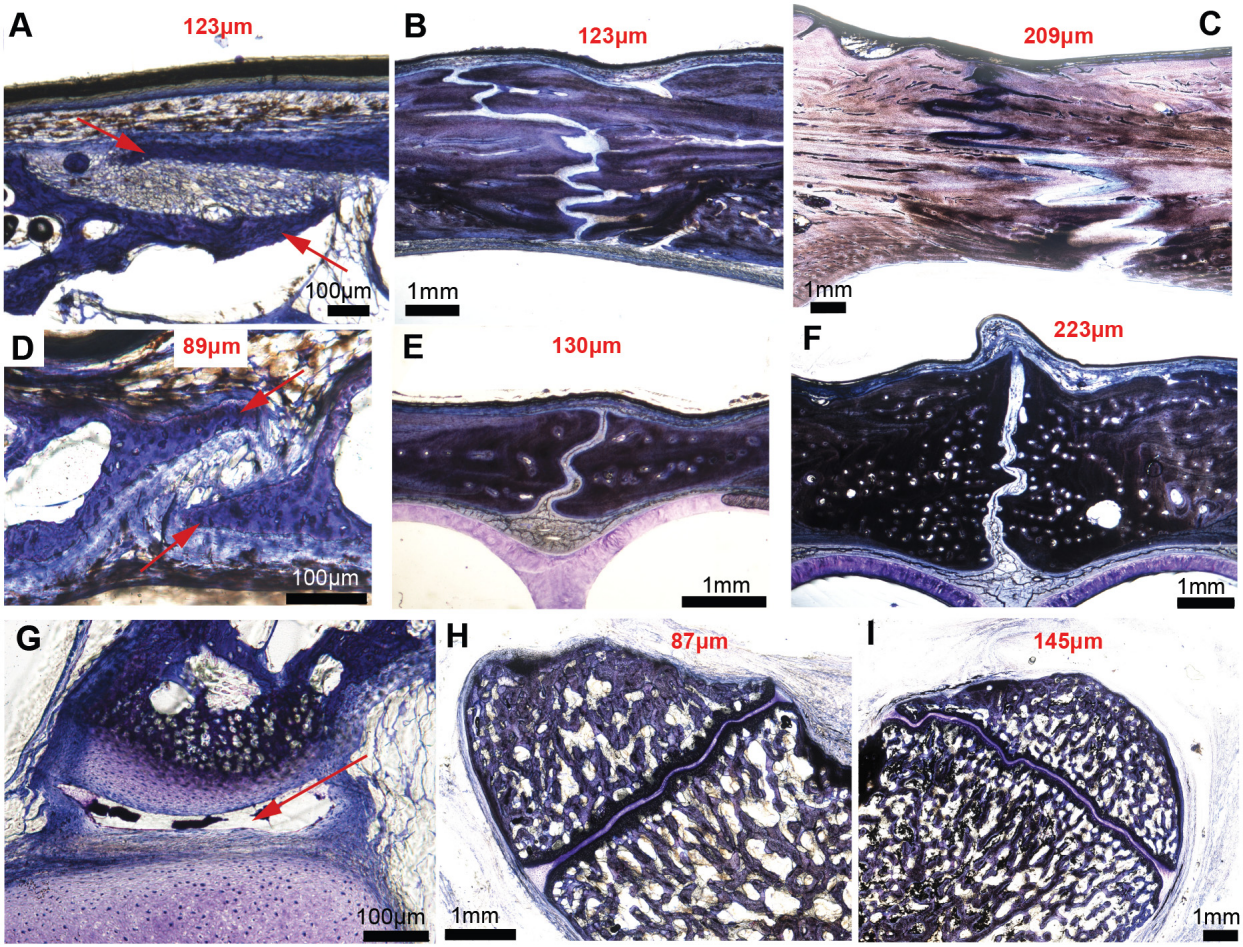
Transverse sections through the frontoparietal suture, the internasal suture and the basioccipital-exoccipital synchondrosis in an ontogenetic series of three American alligator heads, stained with Toluidine-blue. **A-C**, Frontoparietal suture; **D-F**, internasal suture; **G-H**, Basioccipital-exoccipital synchondrosis. The youngest specimen MOR OST 1647 is a few days old and is shown in the first column (A, D, G); the second column shows a 4 to 5 year-old specimen MOR OST 1797 (B, E, H); the third column shows a 9 to 12 year-old sexually mature specimen, MOR OST 1798 (C, F, I). Sutures of the youngest specimen show two thin bone struts overlapping each other (A, D, red arrows). The basioccipital-exoccipital synchondrosis (G) in this same specimen is not fully formed (and appears to have a synovial cavity, red arrow). Interdigitations increase drastically in the frontoparietal suture, but only slightly in the internasal suture (being a midline suture) and in the basioccipital-exoccipital synchondrosis. These sections do not show any sutural bony fusion. The average sutural and synchondroseal widths are marked in red on each photomicrograph, and it is clear that these widths increase as ontogeny progresses.

Histological sampling of one of the sutures that was found to consistently obliterate in emus (the frontoparietal suture; Fig 3) reveals sutural borders that are advancing and an unossified sutural gap becoming progressively narrower through ontogeny (Fig 7A and 7B). The bone fronts ultimately come into contact and the suture becomes completely obliterated (Fig 7C). This trend of progressive sutural obliteration in emus (Figs 5 and 7) is not unexpected, nor counter-intuitive. Moreover, all extant birds appear to present this pattern (e.g., [9,10]). What is counter-intuitive is the pattern presented by the alligators (Fig 6). Fig 8 shows the frontoparietal (Fig 8A–8C) and the internasal sutures (Fig 8D–8F), as well as the basioccipital-exoccipital synchondrosis (Fig 8G–8I) in an ontogenetic series of three American alligator heads (see Table 4 for the age of the specimens). In all of these sections, the sutural borders never come into contact (i.e., they are never fused), even in the oldest specimens (Fig 8C, 8F and 8I). As it was observed morphologically on the surface of the skull, the degree of interdigitation increases generally through ontogeny at the microscopic scale. This is obvious in the frontoparietal suture (Fig 8A–8C) but less in the internasal suture (because midline sutures tend to be relatively straight; Fig 8D–8F) and in the synchondrosis (Fig 8G–8I). We note however that in two larger American alligator skulls, the number and the length of interdigitations are significantly higher and larger than the ones presented here, for all three sampled articulations (*data not shown*, [50]).

The average sutural width (Fig 8A–8F) and synchondroseal width (Fig 8H and 8I) were calculated for each section and are indicated in red (see Fig 2 for the method and Table 5 for full measurements). We note that the average width does increase during ontogeny in each sampled articulation: the frontoparietal suture starts out with a width of 123μm (Fig 8A) and ends at 209μm (Fig 8C); the internasal suture starts at 89μm (Fig 8D) and ends at 223μm (Fig 8F); the basioccipital-exoccipital synchondrosis starts at 87μm (Fig 8H) and ends at 145μm (Fig 8I). The average synchondroseal width could not be calculated in the youngest alligator because, to our surprise, this synchondrosis was not fully formed yet (and resembled a synovial joint with a synovial cavity; Fig 8G, red arrow). These histomorphometric results confirm that the sutures and synchondroses of American alligators do become wider during ontogeny and that the pattern seen in Fig 6 is a biological signal (or at least partly biological, see further elaboration in the Discussion). If larger heads from skeletally mature specimens were histologically sampled, it is highly probable that the sutural gap measurements would be higher than the ones shown here.

**Table 5:** Histomorphometric measurements on American alligator heads.

**Frontoparietal suture**	**Area (μm2)**	**Length (μm)**	**Average Width (μm)**	**Normalized Width**
MOR OST 1647	83871	681	**123**	4.92 (x10^-3^)
MOR OST 1797	870966	7051	**123**	0.93 (x10^-3^)
MOR OST 1798	6412890	30749	**209**	0.73 (x10^-3^)
**Internasal suture**	**Area (μm2)**	**Length (μm)**	**Average Width (μm)**	
MOR OST 1647	25806	290	**89**	3.56 (x10^-3^)
MOR OST 1797	225806	1737	**130**	0.84 (x10^-3^)
MOR OST 1798	1070966	4811	**223**	0.78 (x10^-3^)
**Basioccipital-exoccipital synchondrosis**	**Area (μm2)**	**Length (μm)**	**Average Width (μm)**	
MOR OST 1647	N/A	N/A	N/A	N/A
MOR OST 1797	406451	4638	**87**	0.56 (x10^-3^)
MOR OST 1798	1445158	9931	**145**	0.51 (x10^-3^)

Measurements could not be taken for the basioccipital-exoccipital articulation in MOR OST 1647 because the synchondrosis was not fully formed. N/A: non-applicable.

In the youngest specimens, the sutural areas are formed of very thin, overlapping bony struts, with a narrow sutural gap (Fig 8A and 8D). This explains why most sutures in juvenile alligators were coded as ‘2’ (i.e., closed) to the naked eye. The absence of Howship’s lacunae on the sutural borders (data not shown, [50]) suggest that the sutures of American alligators stay open during ontogeny via some mechanisms that inhibit fusion, rather than by active bone resorption (i.e., a mechanism not uncommon in mammalian sutures e.g., [56,57]).

## Discussion

### Progressive sutural closure: the rule or the exception?

The assessment of maturity in non-avian dinosaur specimens is critical for deciphering the systematics and paleobiology of this group [25,28,42,58]. Moreover, it is crucial to know whether the variation between specimens is ontogenetic or taxonomic in order to have the most accurate estimation of species diversity through time. Determination of ontogenetic stages with quantitative methods using vertebral cartilaginous articulations (synchondroses) as a proxy has already been introduced by two recent pioneering investigations on archosaurs [28,42]. In the present study, by morphologically examining the skull sutures of *D*. *novaehollandiae* and *A*. *mississippiensis*, we find that progressive sutural closure is an accurate proxy for maturity in the former, but not in *Alligator*. The results presented by the American alligator contradict the general assumption that the sutures of all archosaurs are open early in development and fuse progressively during ontogeny. Their sutures do not only stay open, but they also become wider during ontogeny. Our histomorphometric analyses (Fig 8 and Table 5) on alligator heads confirm that the enigmatic pattern of progressive sutural widening shown in Fig 6 is a biological signal. We note however that this sutural widening may be ‘exacerbated’ by skeletonization and thus the degree of widening events may appear more drastic than what they are *in vivo*. As noted earlier, we aimed to minimize this possibility by limiting the use of macerated specimens (see Materials and Methods). Since the American alligator results contradict the general assumption regarding archosaur sutures, by extrapolation, it is safe to infer that progressive sutural closure might not be an accurate maturity indicator in all dinosaurs. Moreover, many other known examples outside of Archosauria are in contradiction with this general assumption used in paleontology: in some extant species, sutures may remain open even well after growth has stopped, while others may obliterate very early as soon as some minimal growth has been reached [5,59]. For example, all the sutures in rats and mice never fuse during their ontogeny (except for the posterior interfrontal suture [60]). Many sutures never close completely in carnivoran mammals as well (the average of closure of 32 sutures across 25 species studied was 28%, which is about 9 sutures out of 32), with the most extreme example being the Northern elephant seal that only shows 14% of closure (approximately 5 sutures out of 32 [61]). The overall suture closure of ruminants is also low, with the majority of species closing less than 25% of their sutures (in that case approximately 7 sutures out of 29 [62]). The most extreme examples would be *Capreolus* (the European roe deer) and *Rubicapra* (the chamois) that only close 5 sutures out of the 29 studied (which corresponds to 17% of closure). Recall that the American alligator showed an average of 5% of closure, and this is much smaller than any number presented in these mammals. Perhaps squamates (and particularly ophidians) would show an even smaller number due to their extremely loose and kinetic skulls related to their feeding adaptations [5].

In the opposite case, some individual sutures or groups of sutures may close very rapidly. For example, the interfrontal suture in humans obliterates between the second and fifth postnatal year [63] (note that the interfrontal suture of the American alligator fuses earlier, during embryonic development). The posterior interfrontal suture of rats and mice also fuses very early, shortly after weaning [60]. Among squamates, closure of synchondroses in the skull base may occur before the animal reaches 30% of its maximum size [58]. This is particularly the case in *Bipes biporus* (the Mexican mole lizard), where the braincase is already closed but almost triples in length until skeletal maturity [58]. Another example not pertaining to the skull but that should still be mentioned here is the case of the caudal vertebrae of *A*. *mississipiensis* that are already fully closed in hatchlings [28]. Moreover, it is known that between closely related clades (or even within clades), the sequences and the amount of sutures that obliterate can be highly variable [3,5,58,61]. For example, all the sutures in the skulls of grizzly bears (*Ursus arctos)* close, while the closely related polar bear (*Ursus maritimus*) only closes half of its sutures [61]. Finally, within a single species, sutural fusion events are sometimes too variable to be reliable for age estimation (e.g., in gray wolves, foxes and humans; see [64–67]).

It is a fact that growth is still possible after the closure of synchondroses (e.g., [28,58]) or even after sutural closure [59], but these “provocative” facts (named as such by Cohen [59]) are rarely mentioned in paleontological studies. The results observed in *A*. *mississippiensis* and the few examples presented above concerning other extant vertebrates suggest that it is incorrect to assume that closed sutures indicate maturity and open sutures indicate juvenescence in Dinosauria (and most likely in other vertebrate clades as well). Consequently, we conclude that the use of sutural closure as a proxy for maturity in extinct archosaurs, including non-avian dinosaurs, should be carefully reconsidered.

### What does sutural widening reflect in *Alligator*?

Not only did *A*. *mississippiensis* contradict the general assumption of a progressive sutural closure through ontogeny (by retaining open sutures), but it also showed an enigmatic pattern of sutural widening. To our knowledge, sutural widening has only been reported in two other species: in the premaxillomaxillary and the nasofrontal sutures of rats ([68]; see the control specimens in their Fig 4A and 4B) and on the ectocranial side of the interfrontal suture of miniature pigs ([55], see their Table 2). Based on these previous observations and on the high number of *Alligator* specimens that were sampled in this study, it is safe to rule out a pathological condition. Pathological conditions related to sutures usually induce their premature fusion, not their widening (i.e., *craniosynostosis*, [59]). Interpreting this pattern of increasing sutural widths in American alligators is very difficult. Sutural widening through ontogeny has been correlated with increased growth rates in mammals [53,55,69]. Therefore, a simple explanation could be that the sutures of alligators get wider with age because their skull size increases. Sutural width has also been correlated to mechanical stresses and stimulation (such as mastication) in mammals [53,54,69,70]. Therefore, another hypothesis is that sutural widening is linked to the feeding habits of American alligators, which undergo shifts in their diet towards progressively larger and more robust, bony prey throughout growth [71,72] (see the estimated diet categories in Fig 6 above the trend line): insects are the predominant food in small individuals from 25 through 60 cm long, while the proportions of crustaceans, fish and reptiles increase as they reach a total length of 122 cm. Up until about 300 cm long, fish are predominant in their diet, and finally the largest alligators (longer than 300 cm) eat mostly large mammals (such as deer and hogs), turtles, and other alligators [71,72]. The bite force of American alligators increases through ontogeny [73] and this, along with some cranial modifications [23], permits access to larger and more robust prey. Biewener proposed that bones and joints maintain constant stress throughout ontogeny [74]. If this is true, perhaps this sutural widening is a mechanism to increase joint size and keep stress constant through ontogeny, since the bite force increases. Therefore, we hypothesize that during the ontogeny of *A*. *mississippiensis*, almost all of its sutures widen in order to accommodate the increasing forces that it receives while feeding on larger and more bony prey items. Sutures are not only sites of bone deposition during growth (i.e., reflecting an ontogenetic signal), but they also determine the biomechanics of the skull in terms of movement and force transmission (reflecting an epigenetic signal [5]). A recent study on the sutures of *Sphenodon* reports that they work collectively to distribute strain throughout its skull [75]. Sutures may stay open during the ontogeny of some species not because their skull is still growing, but because the sutural ligament can reduce the magnitude of stress in cranial bones and can prevent them from fracturing [11]. This has been observed in living species with head-butting behavior (*Ovis orientalis* and *Capra hircus* [76–78]) and during mastication and rooting in miniature pigs (*Sus scrofa* [11]). Moreover, it has been shown that *Tyrannosaurus rex* and *Allosaurus fragilis* retained open sutures throughout ontogeny to accommodate stress and strain during their proposed puncture-pull feeding habits [79–81]. *A*. *mississippiensis* may represent another example of a species where the degree of closure of its sutures reflects biomechanical signals rather than solely ontogenetic signals. Also note that the sutural interdigitations of alligators most likely play a role in stress accommodation: interdigitations are known to stiffen the skull, absorb strain energy and dampen compressive impact forces more effectively than straight sutures [5,82] and normal cranial bone [78]. Both morphologically and histologically, the degree of sutural interdigitation increases during the ontogeny of *A*. *mississippiensis (*but it does not in *D*. *novaehollandiae;* Figs 7 and 8; S3 Fig) and this suggests that even though the sutures of American alligators get wider with age (which could hypothetically weaken the skull and allow less bite force to be delivered to the prey, e.g., see discussion in [80]), they also get stiffer.

An observation which appears to support the hypothesis that sutural widening is related to feeding and increasing bite forces is that generally, the skulls of domestic alligators present sutures that are more open (i.e., they are wider, with a lower average of closure, see the open circles usually below the trend line in Fig 6) than those of wild alligators of the same skull size. Although we have not tested if the apparent differences between wild and domestic alligators of the same skull length are statistically significant, these differences correlate well with previous bite force findings that report that domestic alligators bite more forcefully than their wild counterparts with respect to jaw length [83]. The relatively broader heads of domestic alligators compared to wild alligators may afford more space for the adductor muscles of their jaws [83]. Consequently, perhaps the higher bite forces found in the heads of domestic alligators may be accommodated by more drastic sutural widenings.

As previously mentioned, interpreting the meaning of sutural widening in *Alligator* is not simple. Additional factors that need to be explored include the matter of sutural width normalization. Indeed, it might be suggested that due to the increase in body size throughout growth, in order for the sutural width data to be informative, it should first be normalized. When we normalize the data by skull length, we note that this dimensionless ratio generally decreases through ontogeny (Table 5, see normalized widths). However, it is clear that skull length is not an appropriate measurement for normalization in this case, as skull length increases much more than does sutural width during ontogeny in American alligators. Finding a proper measurement for the normalization of our data is beyond the scope of this paper, if not irrelevant altogether. Our focus was to demonstrate that sutures can widen with age *contra* the assumption that sutures only get narrower during ontogeny (in absolute width with age). The causes and implications of sutural widening during natural development need to be further documented and analyzed in additional extant species (including in archosaurs).

Our hypotheses are still preliminary and need to be tested with three-dimensional biomechanical models and finite-element analyses. However, they are still important for the field of dinosaur paleontology, especially concerning the problem of maturity assessment.

### Implications for Maturity Assessment in Non-Avian Dinosaurs

We have shown that the sutures of *A*. *mississippiensis* do not follow the expected pattern of progressive sutural closure that has been assumed in dinosaur paleontology for decades. Since it is erroneous to assume that open sutures indicate juvenescence and closed sutures indicate maturity in American alligators, by extrapolation, it is possible that the degree of closure of sutures does not reflect ontogenetic statuses accurately in other archosaur species, including non-avian dinosaurs. The degree of sutural closure (or the sutural widths) observed on the skull surface could reflect cranial biomechanics or phylogeny, rather than solely ontogeny. The results of our analyses of emu skulls, on the other hand, suggest the possibility that the degree of sutural closure reflects ontogenetic stages more accurately in dinosaurs closest to the line of birds, since it is a characteristic of all extant birds to synostose their sutures around skeletal maturity, e.g., [9,10]. While suture closure events seem to be very variable and clade-specific within Archosauria, perhaps a more conserved archosaurian pattern of sutural closure exists and could be used for maturity assessment in dinosaur paleontology (see Results section), and this needs to be further investigated.

## Conclusions and Perspectives

Seventy-five years ago, Brown and Schlaikjer suggested that sutural fusion was too variable to be used as a proxy for maturity in the ceratopsian dinosaur *Protoceratops* [84]. The present study suggests that sutural fusion patterns may be tied (at least partly) to species-specific biomechanics and invites reassessments of maturity in other archosaur taxa. Our results are inconsistent with a "rule" of progressive fusion (or progressive sutural narrowing) throughout growth and this suggests that the use of sutural fusion as a proxy for maturity in non-avian dinosaurs should be carefully reconsidered, and even avoided in some instances. We do not advocate the complete abandonment of using sutural fusion as a proxy for maturity, especially if it is employed on dinosaur species with a relatively complete growth series (and for which sutural patterns are understood), but we urge paleontologists to proceed with extreme caution, for example when dealing with an isolated specimen of a yet unknown species. Basic life history characteristics (such as those linked to cranial mechanics) should be assessed prior to employing this method of maturity assessment. Moreover, whenever possible, histological or computed tomography analyses should complement morphological observations. As of today, limb-bone paleohistology remains the most robust single method of maturity assessment in the Dinosauria e.g., [85]. Examinations of sutural fusion patterns in the skulls of other vertebrate taxa (as well as in heads with intact soft-tissues whenever possible) will continue to quantify the degree to which skeletal fusion is indicative of ontogenetic or biomechanical factors.

## Supporting Information

S1 Fig Strict Consensus Phylogenetic Tree for *A*. *mississippiensis*. The numbers (1 through 4) following each museum specimen number indicate ontogenetic categories: 1 for juveniles, 2 for sub-adults, 3 for sexually mature adults and 4 for skeletally mature adults.
(TIF)

S2 Fig Relationship between ontogeny and the averaged degree of sutural closure in the four different anatomical groups of sutures in *D*. *novaehollandiae* and *A*. *mississippiensis*. In the emus, the braincase synchondroses are the first to be completely obliterated (at skeletal maturity), while cranial sutures reach their highest degree of closure later, around the onset of sexual maturity. The least open sutures are the palatal sutures. The degree of closure of facial sutures is intermediate between these two extremes. B) In the alligators, cranial sutures are always more closed than the sutures of any other anatomical group. They are followed by the braincase synchondroses, facial sutures and finally palatal sutures. This ‘hierarchy’ of closure is similar in both emus and American alligators. Abbreviations: Skel mat., skeletally mature adults; Sex. mat., sexually mature adults.
(TIF)

S3 Fig Relationship between ontogeny and the averaged degree of interdigitation in *D*. *novaehollandiae* and *A*. *mississippiensis*. In *A*. *mississippiensis*, the degree of interdigitation increases drastically as ontogeny proceeds. In emus, values are much lower (meaning that sutures are more straight than in the alligators overall) and they increase until sub-adulthood. They are followed by a decrease until sexual maturity, but it is an artifact of the coding used in the phylogenetic analysis. The ‘real’ trend should show an increase of interdigitation followed by a plateau after sub-adulthood in the emus.
(TIF)

S1 File Supporting Information. Character list for the sutures and synchondroses of *D*. *novaehollandiae* and *A*. *mississippiensis* **(Text A).** Taxon-character matrix for *D*. *novaehollandiae* **(Dataset A).** Taxon-character matrix for *A*. *mississippiensis* **(Dataset B).** Sutural closure scores and averages for *D*. *novaehollandiae* **(Table A).** Sutural closure scores and averages for *A*. *mississippiensis* **(Table B).** Sutural interdigitation scores and averages for *D*. *novaehollandiae* **(Table C).** Sutural interdigitation scores and averages for *A*. *mississippiensis* **(Table D).**
(DOCX)
