# Supplementary figures and images for: Fusion Patterns in the Skulls of Modern Archosaurs Reveal That Sutures Are Ambiguous Maturity Indicators for the Dinosauria
Source: PLoS One. 2016 Feb 10;11(2):e0147687. doi: 10.1371/journal.pone.0147687 (PMC4749387; doi:10.1371/journal.pone.0147687)

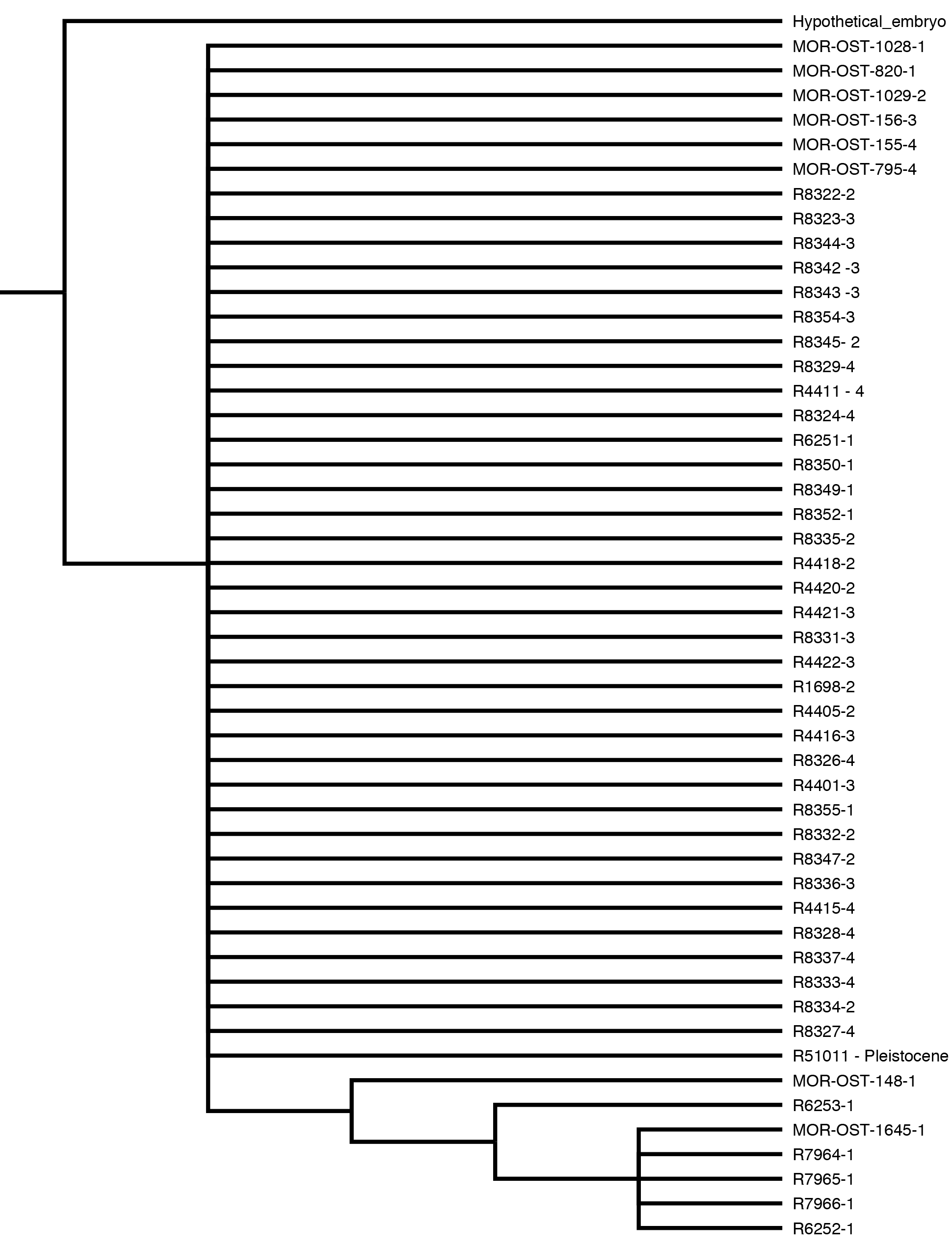

Supplement: S1 Fig — The numbers (1 through 4) following each museum specimen number indicate ontogenetic categories: 1 for juveniles, 2 for sub-adults, 3 for sexually mature adults and 4 for skeletally mature adults. (TIF) [file pone.0147687.s001.tif]

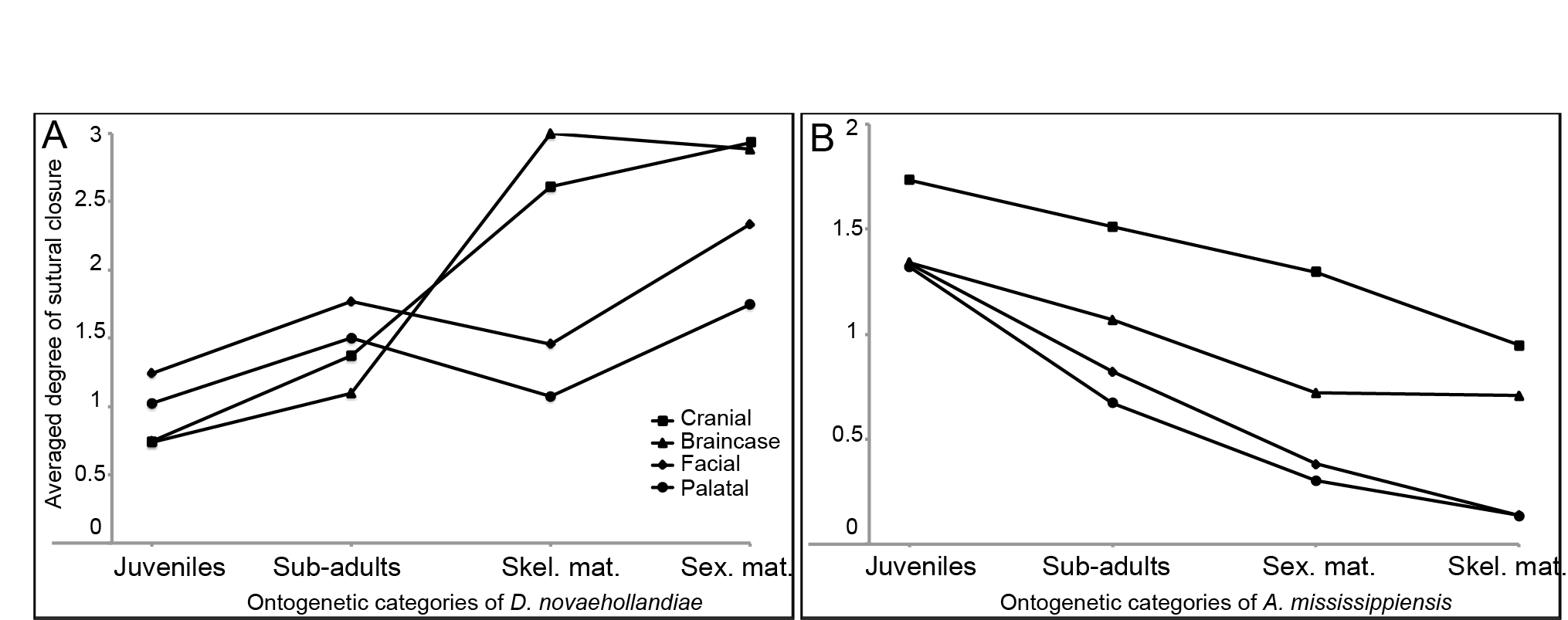

Supplement: S2 Fig — In the emus, the braincase synchondroses are the first to be completely obliterated (at skeletal maturity), while cranial sutures reach their highest degree of closure later, around the onset of sexual maturity. The least open sutures are the palatal sutures. The degree of closure of facial sutures is intermediate between these two extremes. B) In the alligators, cranial sutures are always more closed than the sutures of any other anatomical group. They are followed by the braincase synchondroses, facial sutures and finally palatal sutures. This ‘hierarchy’ of closure is similar in both emus and American alligators. Abbreviations: Skel mat., skeletally mature adults; Sex. mat., sexually mature adults. (TIF) [file pone.0147687.s002.tif]

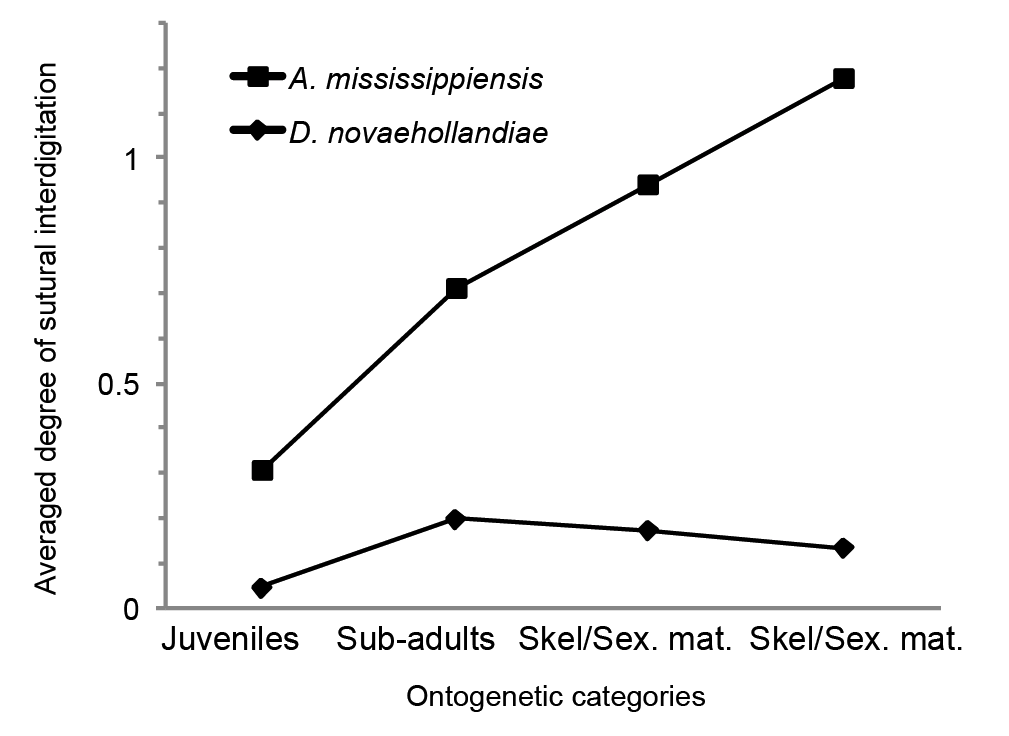

Supplement: S3 Fig — In A. mississippiensis, the degree of interdigitation increases drastically as ontogeny proceeds. In emus, values are much lower (meaning that sutures are more straight than in the alligators overall) and they increase until sub-adulthood. They are followed by a decrease until sexual maturity, but it is an artifact of the coding used in the phylogenetic analysis. The ‘real’ trend should show an increase of interdigitation followed by a plateau after sub-adulthood in the emus. (TIF) [file pone.0147687.s003.tif]
