# Supplementary material for: Fusion Patterns in the Skulls of Modern Archosaurs Reveal That Sutures Are Ambiguous Maturity Indicators for the Dinosauria
Source: PLoS One. 2016 Feb 10;11(2):e0147687. doi: 10.1371/journal.pone.0147687 (PMC4749387; doi:10.1371/journal.pone.0147687)
Supplement: S1 File — Character list for the sutures and synchondroses of D. novaehollandiae and A. mississippiensis (Text A). Taxon-character matrix for D. novaehollandiae (Dataset A). Taxon-character matrix for A. mississippiensis (Dataset B). Sutural closure scores and averages for D. novaehollandiae (Table A). Sutural closure scores and averages for A. mississippiensis (Table B). Sutural interdigitation scores and averages for D. novaehollandiae (Table C). Sutural interdigitation scores and averages for A. mississippiensis (Table D). (DOCX) [file pone.0147687.s004.docx]

**Supporting Information**

**S1 File. Supporting Information.** Character list for the sutures and synchondroses of *D. novaehollandiae* and *A. mississippiensis* **(Text A).** Taxon-character matrix for *D. novaehollandiae* **(Dataset A).** Taxon-character matrix for *A. mississippiensis* **(Dataset B).** Sutural closure scores and averages for *D. novaehollandiae* **(Table A).** Sutural closure scores and averages for *A. mississippiensis* **(Table B).** Sutural interdigitation scores and averages for *D. novaehollandiae* **(Table C).** Sutural interdigitation scores and averages for *A. mississippiensis* **(Table D).**

**S1 Fig. Strict Consensus Phylogenetic Tree for *A. mississippiensis.***

The numbers (1 through 4) following each museum specimen number indicate ontogenetic categories: 1 for juveniles, 2 for sub-adults, 3 for sexually mature adults and 4 for skeletally mature adults.

**S2 Fig. Relationship between ontogeny and the averaged degree of sutural closure in the four different anatomical groups of sutures in *D. novaehollandiae* and *A. mississippiensis*.**

In the emus, the braincase synchondroses are the first to be completely obliterated (at skeletal maturity), while cranial sutures reach their highest degree of closure later, around the onset of sexual maturity. The least open sutures are the palatal sutures. The degree of closure of facial sutures is intermediate between these two extremes. B) In the alligators, cranial sutures are always more closed than the sutures of any other anatomical group. They are followed by the braincase synchondroses, facial sutures and finally palatal sutures. This ‘hierarchy’ of closure is similar in both emus and American alligators. Abbreviations: Skel mat., skeletally mature adults; Sex. mat., sexually mature adults.

**S3 Fig. Relationship between ontogeny and the averaged degree of interdigitation in *D. novaehollandiae* and *A. mississippiensis*.**

In *A. mississippiensis*, the degree of interdigitation increases drastically as ontogeny proceeds. In emus, values are much lower (meaning that sutures are more straight than in the alligators overall) and they increase until sub-adulthood. They are followed by a decrease until sexual maturity, but it is an artifact of the coding (the question marks) used in the phylogenetic analysis. The ‘real’ trend should show an increase of interdigitation followed by a plateau after sub-adulthood in the emus.

**S1 Text. Character List for the Sutures and Synchondroses of *D. novaehollandiae* and *A. mississippiensis***

Character List for the Sutures and Synchondroses of *D. novaehollandiae*

Character 1: nasal-mesethmoid: (0) open; (1) partially closed; (2) closed (but still visible), (3) completely obliterated

Character 2: nasal-mesethmoid: (0) straight; (1) interdigitated; (2) very interdigitated

Character 3: nasal-prefrontal: (0) open; (1) partially closed; (2) closed (but still visible), (3) completely obliterated

Character 4: nasal-prefrontal: (0) straight; (1) interdigitated; (2) very interdigitated

Character 5: nasal-frontal: (0) open; (1) partially closed; (2) closed (but still visible), (3) completely obliterated

Character 6: nasal-frontal: (0) straight; (1) interdigitated; (2) very interdigitated

Character 7: interfrontal: (0) open; (1) partially closed; (2) closed (but still visible), (3) completely obliterated

Character 8: interfrontal: (0) straight; (1) interdigitated; (2) very interdigitated

Character 9: frontal-parietal: (0) open; (1) partially closed; (2) closed (but still visible), (3) completely obliterated

Character 10: frontal-parietal: (0) straight; (1) interdigitated; (2) very interdigitated

Character 11: interparietal: (0) open; (1) partially closed; (2) closed (but still visible), (3) completely obliterated

Character 12: interparietal: (0) straight; (1) interdigitated; (2) very interdigitated

Character 13:supraoccipital-parietal: (0) open; (1) partially closed; (2) closed (but still visible), (3) completely obliterated

Character 14: supraoccipital-parietal: (0) straight; (1) interdigitated; (2) very interdigitated

Character 15: exoccipital-squamosal (0) open; (1) partially closed; (2) closed (but still visible), (3) completely obliterated

Character 16: exoccipital-squamosal: (0) straight; (1) interdigitated; (2) very interdigitated

Character 17: exoccipital-supraoccipital: (0) open; (1) partially closed; (2) closed (but still visible), (3) completely obliterated

Character 18: exoccipital-supraoccipital: (0) straight; (1) interdigitated; (2) very interdigitated

Character 19: exoccipital basioccipital: (0) open; (1) partially closed; (2) closed (but still visible), (3) completely obliterated

Character 20: exoccipital-basioccipital: (0) straight; (1) interdigitated; (2) very interdigitated Character 21: premaxillo-nasal patency: (0) open; (1) partially closed; (2) closed (but still visible), (3) completely obliterated

Character 22: premaxillo-nasal: (0) straight; (1) interdigitated; (2) very interdigitated

Character 23: frontal-mesethmoid: (0) open; (1) partially closed; (2) closed (but still visible), (3) completely obliterated

Character 24: frontal-mesethmoid: (0) straight; (1) interdigitated; (2) very interdigitated

Character 25: parietal-squamosal patency: (0) open; (1) partially closed; (2) closed (but still visible), (3) completely obliterated

Character 26: parietal-squamosal: (0) straight; (1) interdigitated; (2) very interdigitated

Character 27: laterosphenoid-parietal patency: (0) open; (1) partially closed; (2) closed (but still visible), (3) completely obliterated

Character 28: Laterosphenoid-parietal: (0) straight; (1) interdigitated; (2) very interdigitated

Character 29: Lateropshenoid-squamosal: (0) open; (1) partially closed; (2) closed (but still visible), (3) completely obliterated

Character 30: Laterosphenoid-squamosal: (0) straight; (1) interdigitated; (2) very interdigitated

Character 31: (Palatal view) Basiparasphenoid-basioccipital: (0) open; (1) partially closed; (2) closed (but still visible), (3) completely obliterated

Character 32: (Palatal view) Basiparasphenoid-basioccipital: (0) straight; (1) interdigitated; (2) very interdigitated

Character 33: (Palatal view) pterygoid-vomer: (0) open; (1) partially closed; (2) closed (but still visible), (3) completely obliterated

Character 34: (Palatal view) pterygoid- vomer: (0) straight; (1) interdigitated; (2) very interdigitated

Character 35: (Palatal view) vomer-palatine patency: (0) open; (1) partially closed; (2) closed (but still visible), (3) completely obliterated

Character 36: (Palatal view) vomer-palatine: (0) straight; (1) interdigitated; (2) very interdigitated

Character 37: (Palatal view) maxilla-premaxilla patency: (0) open; (1) partially closed; (2) closed (but still visible), (3) completely obliterated

Character 38: (Palatal view) maxilla-premaxilla: (0) straight; (1) interdigitated; (2) very interdigitated

Character 39: (Palatal view) premaxilla-vomer: (0) open; (1) partially closed; (2) closed (but still visible), (3) completely obliterated

Character 40: (Palatal view) premaxilla-vomer : (0) straight; (1) interdigitated; (2) very interdigitated

Character 41: (Dorsal view) Maxilla-premaxilla patency: (0) open; (1) partially closed; (2) closed (but still visible), (3) completely obliterated

Character 42: (Dorsal view) Maxilla-premaxilla: (0) straight; (1) interdigitated; (2) very interdigitated

Character List for the Sutures and Synchondroses of *A. mississippiensis*

Character 1: interpremaxillary: (0) open; (1) partially closed; (2) closed (but still visible); (3) completely obliterated

Character 2: interpremaxillary: (0) straight; (1) interdigitated; (2) very interdigitated

Character 3: premaxilla-maxilla: (0) open; (1) partially closed; (2) closed (but still visible); (3) completely obliterated

Character 4: premaxilla-maxilla:(0) straight; (1) interdigitated; (2) very interdigitated

Character 5: premaxilla-nasal: (0) open; (1) partially closed; (2) closed (but still visible); (3) completely obliterated

Character 6: premaxilla-nasal: (0) straight; (1) interdigitated; (2) very interdigitated

Character 7: internasal: (0) open; (1) partially closed; (2) closed (but still visible); (3) completely obliterated

Character 8: internasal: (0) straight; (1) interdigitated; (2) very interdigitated

Character 9: nasal-maxilla: (0) open; (1) partially closed; (2) closed (but still visible); (3) completely obliterated

Character 10: nasal-maxilla: (0) straight; (1) interdigitated; (2) very interdigitated

Character 11: nasal-prefrontal: (0) open; (1) partially closed; (2) closed (but still visible); (3) completely obliterated

Character 12: nasal-prefrontal: (0) straight; (1) interdigitated; (2) very interdigitated

Character 13: prefrontal-lachrymal: (0) open; (1) partially closed; (2) closed (but still visible); (3) completely obliterated

Character 14: prefrontal-lachrymal: (0) straight; (1) interdigitated; (2) very interdigitated

Character 15: maxilla-lachrymal: (0) open; (1) partially closed; (2) closed (but still visible); (3) completely obliterated

Character 16: maxilla-lachrymal: (0) straight; (1) interdigitated; (2) very interdigitated

Character 17: lachrymal-jugal: (0) open; (1) partially closed; (2) closed (but still visible); (3) completely obliterated

Character 18: lachrymal-jugal: (0) straight; (1) interdigitated; (2) very interdigitated

Character 19: maxilla-jugal: ((0) open; (1) partially closed; (2) closed (but still visible); (3) completely obliterated

Character 20: maxilla-jugal: (0) straight; (1) interdigitated; (2) very interdigitated

Character 21: frontal-prefrontal: ((0) open; (1) partially closed; (2) closed (but still visible); (3) completely obliterated

Character 22: frontal-prefrontal: (0) straight; (1) interdigitated; (2) very interdigitated

Character 23: nasal-frontal: (0) open; (1) partially closed; (2) closed (but still visible); (3) completely obliterated

Character 24: nasal-frontal: (0) straight; (1) interdigitated; (2) very interdigitated

Character 25: interfrontal: (0) open; (1) partially closed; (2) closed (but still visible); (3) completely obliterated

Character 26: interfrontal: (0) straight; (1) interdigitated; (2) very interdigitated

Character 27: frontal-parietal: (0) open; (1) partially closed; (2) closed (but still visible); (3) completely obliterated

Character 28: frontal-parietal: (0) straight; (1) interdigitated; (2) very interdigitated

Character 29: interparietal: (0) open; (1) partially closed; (2) closed (but still visible); (3) completely obliterated

Character 30: interparietal: (0) straight; (1) interdigitated; (2) very interdigitated

Character 31: parietal-squamosal: (0) open; (1) partially closed; (2) closed (but still visible); (3) completely obliterated

Character 32: parietal-squamosal: (0) straight; (1) interdigitated; (2) very interdigitated

Character 33: frontal-postorbital: (0) open; (1) partially closed; (2) closed (but still visible); (3) completely obliterated

Character 34: frontal-postorbital: (0) straight; (1) interdigitated; (2) very interdigitated

Character 35: postorbital-squamosal: (0) open; (1) partially closed; (2) closed (but still visible); (3) completely obliterated

Character 36: postorbital-squamosal: (0) straight; (1) interdigitated: (2) very interdigitated

Character 37: (palatal view) premaxilla-maxilla: (0) open; (1) partially closed; (2) closed (but still visible); (3) completely obliterated

Character 38: (palatal view) premaxilla-maxilla: (0) straight; (1) interdigitated; (2) very interdigitated

Character 39: (palatal view) intermaxillary: (0) open; (1) partially closed; (2) closed (but still visible); (3) completely obliterated

Character 40: (palatal view) intermaxillary (0) straight; (1) interdigitated; (2) very interdigitated

Character 41: (palatal view) maxilla-palatine: (0) open; (1) partially closed; (2) closed (but still visible); (3) completely obliterated

Character 42: (palatal view) maxilla-palatine: (0) straight; (1) interdigitated; (2) very interdigitated

Character 43: (palatal view) interpalatine: (0) open; (1) partially closed; (2) closed (but still visible); (3) completely obliterated

Character 44: (palatal view) interpalatine: (0) straight; (1) interdigitated; (2) very interdigitated

Character 45: supraoccipital-parietal: (0) open; (1) partially closed; (2) closed (but still visible); (3) completely obliterated

Character 46: supraoccipital-parietal: (0) straight; (1) interdigitated; (2) very interdigitated

Character 47: exoccipital-squamosal (0) open; (1) partially closed; (2) closed (but still visible); (3) completely obliterated

Character 48: exoccipital-squamosal: (0) straight; (1) interdigitated; (2) very interdigitated

Character 49: exoccipital-supraoccipital: (0) open; (1) partially closed; (2) closed (but still visible); (3) completely obliterated

Character 50: exoccipital-supraoccipital: (0) straight; (1) interdigitated; (2) very interdigitated

Character 51: exoccipital-basioccipital: (0) open; (1) partially closed; (2) closed (but still visible); (3) completely obliterated

Character 52: exoccipital-basioccipital: (0) straight; (1) interdigitated; (2) very interdigitated

Character 53: (endocranial view) parietal-supraoccipital : (0) open; (1) partially closed; (2) closed (but still visible); (3) completely obliterated

Character 54: (endocranial view) parietal-supraoccipital : (0) straight, (1) interdig, (2) very interdigitated

Character 55: (endocranial view) parietal-frontal: (0) open; (1) partially closed; (2) closed (but still visible); (3) completely obliterated

Character 56: (endocranial view) parietal-frontal: (0) straight, (1) interdig, (2) very interdigitated

Character 57: (medial view) laterosphenoid-prootic: (0) open; (1) partially closed; (2) closed (but still visible); (3) completely obliterated

Character 58: (medial view) laterosphenoid-prootic: (0) straight; (1) interdigitated; (2) very interdigitated

Character 59: (medial view) laterosphenoid-basisphenoid: (0) open; (1) partially closed; (2) closed (but still visible); (3) completely obliterated

Character 60: (medial view) laterosphenoid-basisphenoid: (0) straight; (1) interdigitated; (2) very interdigitated

Character 61: (medial view) basisphenoid-prootic: (0) open; (1) partially closed; (2) closed (but still visible); (3) completely obliterated

Character 62: (medial view) basisphenoid-prootic: (0) straight; (1) interdigitated; (2) very interdigitated

Character 63: (medial view) exoccipital-prootic: (0) open; (1) partially closed; (2) closed (but still visible); (3) completely obliterated

Character 64: (medial view) exoccipital-prootic: (0) straight; (1) interdigitated; (2) very interdigitated

Character 65: (medial view) exoccipital-basioccipital: (0) open; (1) partially closed; (2) closed (but still visible); (3) completely obliterated

Character 66: (medial view) exoccipital-basioccipital: (0) straight; (1) interdigitated; (2) very interdigitated

Character 67: (medial view) basioccipital-basisphenoid: (0) open; (1) partially closed; (2) closed (but still visible); (3) completely obliterated

Character 68: (medial view) basioccipital-basisphenoid: (0) straight; (1) interdigitated; (2) very interdigitated

Character 69: (lateral view) laterosphenoid-pterygoid: (0) open; (1) partially closed; (2) closed (but still visible); (3) completely obliterated

Character 70: (lateral view) laterosphenoid-pterygoid: (0) straight; (1) interdigitated; (2) very interdigitated

Character 71: L(lateral view) laterosphenoid-quadrate: (0) open; (1) partially closed; (2) closed (but still visible); (3) completely obliterated

Character 72: (lateral view) laterospehnoid-quadrate: (0) straight; (1) interdigitated; (2) very interdigitated

Character 73: (lateral view) quadrate-pterygoid: (0) open; (1) partially closed; (2) closed (but still visible); (3) completely obliterated

Character 74: (lateral view) quadrate-pterygoid: (0) straight; (1) interdigitated; (2) very interdigitated

Character 75: (ventro-caudal view) basisphenoid-pterygoid: (0) open; (1) partially closed; (2) closed (but still visible); (3) completely obliterated

Character 76: (ventro-caudal view) basisphenoid-pterygoid: (0) straight; (1) interdigitated; (2) very interdigitated

Character 77: (ventro-caudal view) basisphenoid-basioccipital: (0) open; (1) partially closed; (2) closed (but still visible); (3) completely obliterated

Character 78: (ventro-caudal view) basisphenoid-basioccipital: (0) straight; (1) interdigitated; (2) very interdigitated

Character 79: (ventro-caudal view) exoccipital-quadrate: (0) open; (1) partially closed; (2) closed (but still visible); (3) completely obliterated

Character 80: (ventro-caudal view) exoccipital-quadrate: (0) straight; (1) interdigitated; (2) very interdigitated

**S1 Dataset. Taxon-character matrix for *D. novaehollandiae***

*D. novaehollandiae*

Hypothetical embryo

0 0 0 0 0 0 0 0 0 0 0 0 0 0 0 0 0 0 0 0 0 0 0 0 0 0 0 0 0 0 0 0 0 0 0 0 0 0 0 0 0 0

MOR-OST-1298

? ? ? ? ? ? 0&1 1 0&1 1 1 1 0&1 0 0 0 0 0 1 0 ? ? ? ? 1 0 1 0 1 0 0&1 0 ? ? ? ? ? ? ? ? ? ?

MOR-OST-1297

1&2 0 2 0 3 ? 2&3 0 3 ? 3 ? 2&3 0 3 ? 3 ? 3 ? ? ? 1 0 2 0 3 ? 3 ? 3 ? ? ? ? ? ? ? ? ? ? ?

MOR-OST-186

1&2 0 3 ? 3 ? 2 0 3 ? 3 ? 2&3 0 3 ? 3 ? 3 ? ? ? 2 0 3 0 3 ? 3 ? 3 ? ? ? ? ? ? ? ? ? ? ?

MOR-OST-232

2 0 3 ? 3 ? 2 0 3 ? 3 ? 2&3 0 3 ? 3 ? 3 ? 1 0 2 0 3 0 3 ? 3 ? 2 0 2 0 1 0 2 1 2 0 2&3 0

ROM R7630

? ? ? ? ? ? ? ? 1 1 ? ? 0&1 0 0 0 0 0 1 0 ? ? ? ? 0 0 0 0 0 0 0 0 0 0 1 0 ? ? ? ? ? ?

ROM R7644

? ? ? ? ? ? 1 0 1 1 1 1 1 0 0 0 1 0 1 0 ? ? ? ? ? ? 1 0 1 0 1 0 ? ? ? ? ? ? ? ? ? ?

ROM R7945

1 0 1 0 0 0 1 0 1 0 1 1 1 0 1 0 1 0 2 0 1 0 1 0 ? ? 1 0 1 0 1 0 1 0 1 0 2 0 1 0 ? ?

ROM R7654

1 0 1 0 2 0 1 1 2 1 2 1 2 0 3 0 3 0 3 0 1 0 1 0 ? ? 3 0 3 0 3 0 1 0 0&1 0 2 0 1 0 2 2

ROM R6843

2 0 3 0 2 0 3 0 3 0 3 0 3 0 3 0 3 0 3 0 1&2 0 2 0 ? ? 3 0 3 0 3 0 2 0 1 0 2 0 2 0 2 0

MOR-OST-1803

3 ? 3 ? 3 ? 3 ? 3 ? 3 ? 3 ? 3 ? 3 ? 3 ? 2 0 3 ? 3 ? 3 ? 3 ? 3 ? 2 1 1 1 2 1 2 1 2 0

MOR-OST-1805

0&1&2 0 2 0 1&2 0 0&1 0 0&1 0 0&1 0 0 0 0 0 0 0 1 0 1 0 0&1&2 0 1 0 ? ? 1 0 0&1 0 1 0 1 0 1&2 0 2 0 1&2 0

MOR-OST-1806

0&1&2 0 2 0 2 0 0&1 1 0&1 0 0&1 0 0&1 0 0 0 0 0 1 0 2 0 2 0 1 0 1 0 1 0 1 0 1 0 ? ? 0&1 0 1 0 0 0

MOR-OST-1807

2 0 2 0 2 0 0&1 1 0&1 0 0&1 0 1&2 0 0 0 1 0 1 0 1 0 2 0 1&2 0 1 0 1 0 1 0 2 0 2 0 0&1 0 0 0 0 0

MOR-OST-1808

2 0 2 0 2 1 1&2 1 2 1 2 1 2 0 1 0&1 1 0 1 0 2 0 2 0 1&2 0 0 0 1 1 1&2&3 0 2 0 ? ? 2 0 2 0 2 1

MOR-OST-1809

2 0 1 0 2 0 1 0 1 0 1 1 1 0 1 0&1 1&2 0 1&2&3 0 1&2 0 1 0 2&3 0 3 ? 2&3 0 1&2&3 0 0 0 0&1 0 2 1 2 0 2 0

MOR-OST-1810

0 0 1 0 1 0 1 1 1 1 1 1 2 0 3 ? 3 ? 3 ? 1 0 1 0 2&3 0 3 ? 3 ? 3 ? 0&1 0 0 0 1 1 0 0 1 0

MOR-OST-1811

2 0 2 0 2 0 2 1 3 ? 3 ? 3 ? 3 ? 3 ? 3 ? 1 0 1 0 3 ? 3 ? 3 ? 3 ? 1&2 0 0 0 2&3 1 2 0 1 0

MOR-OST-1812

1 0 1&2 0 2&3 0 1 0 2&3 0 3 ? 3 ? 3 ? 3 ? 3 ? 0 0 1 0 3 ? 3 ? 3 ? 3 ? 1&2 0 0 0 1 1 1 0 1 0

MOR-OST-1813

1 0 1&2 0 2&3 0 1&2 0 3 ? 3 ? 2&3 0 3 ? 3 ? 3 ? 1&2 0 1 0 3 ? 3 ? 3 ? 3 ? 1&2 0 0&1 0 1 1 1 0 1 0

MOR-OST-1814

1&2 0 1 0 2 0 1 0 1&2&3 1 3 ? 3 ? 3 ? 3 ? 3 ? 1&2 0 1 0 3 ? 3 ? 3 ? 3 ? 1 0 1 0 1&2 1 1 0 2 0

MOR-OST-1815

2 1 1 0 2 0 1 0 1&2&3 1 3 ? 3 ? 3 ? 3 ? 3 ? 1&2 0 1 0 3 ? 3 ? 3 ? 3 ? 1 0 1 0 2 1 2 0 2 0

MOR-OST-1800

2 0 2 0 2 0 1&2 0 0&1&2 1 0&2 1 2 0 1 0 1 0 1 0 1&2 0 2 0 2 0 2 0 2 0 0&1 0 1 0 ? ? 2 1 2 0 2 0

MOR-OST-1802

1 0 1&2 0 2 0 1&2 0 1&2 1 2 1 2 0 2 0 1 0 1 0 2 0 2 0 2 0 1 0 1 0 1 0 ? ? ? ? 1&2 1 1 0 1 0

MOR-OST-1799

0 0 2 0 2 0 0&1 0 0&1&2 0 0 0 0&1 0 0&1 0 0 0 1 0 2 0 0 0 2 0 2 0 2 0 0 0 2 0 1 0 0 0 0 0 0 0

**S2 Dataset. Taxon-character matrix for *A. mississippiensis***

Hypothetical embryo

0 0 0 0 0 0 0 0 0 0 0 0 0 0 0 0 0 0 0 0 0 0 0 0 0 0 0 0 0 0 0 0 0 0 0 0 0 0 0 0 0 0 0 0 0 0 0 0 0 0 0 0 0 0 0 0 0 0 0 0 0 0 0 0 0 0 0 0 0 0 0 0 0 0 0 0 0 0 0 0

MOR-OST-1645

? ? 1 0 2 0 2 0 2 0 2 0 2 0 2 0 2 0 1 0 ? 0 1 0 3 ? 1 0 1&2&3 ? 1 0 1 1 ? ? 2 0 1 0 1 0 2 0 1 0 1 1 0&1 0 1 0 ? ? ? ? ? ? ? ? ? ? ? ? ? ? ? ? 0 0 0 0 1 0 0 0 1 0 1 0

MOR-OST-148

2 0 1 1 2 0 2 0 1 0 2 0 2 0 1 0 1 0 1&2 0 2 0 1 0 3 ? 1 0 3 ? 2 0 2 0 1 1 2 0 2 0 1 1 1 0 2 0 2 0 2 0 1 0 ? ? ? ? ? ? ? ? ? ? ? ? ? ? ? ? 2 0 2 0 2 1 2 1 0 0 2 0

MOR-OST-1028

1 0 1 1 1 0 2 0 1 0 1 0 1 0 1 0 2 0 1 0 0 1 1 1 3 ? 0 1 3 ? 1 1 2 0 0 1 2 1 1 0 1 0 2 0 1 1 2 1 2 0 1 0 ? ? ? ? ? ? ? ? ? ? ? ? ? ? ? ? 2 1 1 0 2 1 2 1 2 0 2 0

MOR-OST-820

1 0 0 1 1 0 1 0 1 0 1 0 1 0 1 1 0 0 0 1 1 1 0 1 3 ? 0 1 3 ? 1 0 2 0 0 1 0 1 1 0 0 1 1 0 2 0 2 1 2 1 1 0&1 ? ? ? ? ? ? ? ? ? ? ? ? ? ? ? ? 0 1 1 1 0 1 1 1 2 2 1&2 0

MOR-OST-1029

0 0 0&1 1 0 0&1 2 0 2 1 1 1 1 0 1 1 1 1 1 1 1 1 0 1 3 ? 1 1 3 ? 1 1 0 1 0 1 0 1 1 1 1 1 1 0 1 1 2 1 1 1 2 0 ? ? ? ? ? ? ? ? ? ? ? ? ? ? ? ? 1 1 1 1 1 1 1 1 0 2 1 0

MOR-OST-156

1 0 0 2 0 2 0&1&2 0 0&1 1 0 1 0 1 0 2 0 1 0 1 0 1 0 1 3 ? 0 1 3 ? 0 1 0 2 0 2 0 2 0 1 0 1 0 0 1 1 0 1 0 1 0 0 ? ? ? ? ? ? ? ? ? ? ? ? ? ? ? ? 0 1 0 1 0&1&2 1 0 2 0 2 0 0

MOR-OST-155

0 0 0 2 0 2 0 1 1 1 0 1 1&2 0 0 1 1 1 1 1 0 1 0 1 3 ? 1 1 3 ? 2 1 2 2 1 2 0 2 0 2 0 1 0 0 1 1 1 1 0 1 1 0 ? ? ? ? ? ? ? ? ? ? ? ? ? ? ? ? 1 1 2 1 2 1 0 2 0 2 0 0

MOR-OST-795

? ? ? 2 ? 2 ? 1 0 1 0 2 0 1 0 2 0 1 0 1 0 2 0 2 3 ? 0 2 3 ? 0 2 0 2 0 2 ? 2 ? ? 0 1 ? ? 1 1 0 1 0 1 0 1 ? ? ? ? ? ? ? ? ? ? ? ? ? ? ? ? 0 2 1 1 1&2 1 0 ? 0 2 0 0

ROM R8322

1 0 1 1 1 1 1 0 1 1 1 0 1 0 1 1 1 0 1 0 1 0 1 1 3 ? ? ? ? ? ? 1 ? 1 1 ? 1 1 1 1 1 1 1 0 0 0 ? ? ? ? 1 1 ? ? ? ? 1 0 1 0 2 1 0 0 1 1 1 1 1 1 2 1 1 1 1 1 0 2 1 1

ROM R8323

? ? ? ? ? 1 1 0 0 0 0 0 0 0 0 1 0 0 0 1 0 1 0 1 3 ? ? ? ? ? ? 1 0 1 0 1 ? 1 0 2 0 1 0 0 ? ? ? ? ? ? 1 1 ? ? ? ? 1 1 0 1 0 1 0 0 1 1 0 1 0 1 1 1 0 1 0 2 0 2 0 1

ROM R8344

0 0 0 1 0 1 1 0 1 0 1 0 1 0 1 1 1 0 0 1 1 1 1 1 ? ? 1 1 ? ? 3 ? 1 1 1 1 1 1 0 1 1 1 0 0 ? ? 1 1 1 1 0 1 ? ? ? ? ? ? ? ? ? ? ? ? ? ? ? ? 1 1 1 1 1 1 1 2 0 2 0 0

ROM R8342

0 1 0 1 1 1 1 1 0 1 1 1 1 0 0 2 1 0 1 1 0 1 1 1 3 ? 1 1 3 ? 3 1 0 1 1 1 0 1 0 1 1 1 1 0 1 0 1 1 1 1 ? ? ? ? ? ? ? ? ? ? ? ? ? ? ? ? ? ? 1 1 2 1 1 1 1 2 0 2 0 1

ROM R8343

? ? 0 2 0 2 0 1 1 1 0 1 0 1 0 2 1 1 1 1 1 1 0 1 3 ? 1 1 3 ? ? ? 1 1 1 1 0 2 0 2 0 2 0 1 2 0 1 1 1 1 0 1 1 1 1 2 1 1 0 1 1 1 0 0 0 1 0 1 2 1 1 1 2 1 1 2 0 2 0 1

ROM R8354

1 0 1 1 1 0 1 0 1 1 1 1 1 1 1 1 1 0 1 1 1 1 1 1 3 ? 1 1 3 ? 1 1 1 1 1 1 1 1 1 0 1 1 1 0 1 0 1 1 ? 0 1 1 ? ? ? ? ? ? ? ? ? ? ? ? ? ? ? ? 2 1 2 0 2 0 1 1 2 0 1 0

ROM R8345

1 0 1 1 1 0 1 0 1 0 1 1 1 1 1 1 1 0 1 1 1 1 1 1 3 ? 1 1 3 ? 1 1 1 1 1 1 1 1 1 0 1 1 1 0 1 1 2 1 ? ? 1 1 ? ? ? ? ? ? ? ? ? ? ? ? ? ? ? ? 1 1 2 0 2 0 1 1 2 0 1 0

ROM R8329

0&1&2 0 0 2 0 2 1 1 1 1 0 1 0 0 0 1 0 1 0 2 1 1 0 1 3 ? 1 1 ? ? 0 1 0 1 0 1 0 2 0 2 1 1 1 1 ? ? 2 1 2 1 1 1 ? ? ? ? 2 0 1 1 2 1 1 0 2 1 2 1 1 1 1 0 2 0 0 2 0 2 0 0

ROM R4411

0 0 0 2 0 1 0 1 0 1 0 1 0 1 0 1 0 1 0 1 0 1 0 1 2&3 ? 0 1 3 ? 0 2 0 2 0 2 0 2 0 2 0 2 0 0 0 1 0 2 1 1 1 1 1 1 0 2 1 1 0 1 0 1 0 0 0 1 0 1 0 1 0 1 1 0 0 2 0 2 0 0

ROM R8324

0 2 0 2 0 1 0 2 0 1 0 1 0 1 0 2 0 1 0 1 0 1 0 2 3 ? 0 1 3 ? 0 1 0 2 0 2 0 2 0 2 0 2 0 1 0 1 0 2 1 1 1 2 ? ? ? ? 1 1 1 1 1 1 0 0 1 0 1 1 1 2 1 1 0 1 0 2 0 2 0 1

ROM R6251

1 0 1 1 2 0 1 0 1 0 0 1 1 0 0 0 0 0 1 0 1 1 2 0 3 ? 1 1 3 ? 2 0 2 1 1 1 2 1 1 0 2 1 1 0 1 0 2 0 1 0 2 0 ? ? ? ? ? ? ? ? ? ? ? ? ? ? ? ? 1 1 2 0 1 0 1 0 2 1 2 0

ROM R8350

1 0 1 1 1 1 1 0 1 0 1 1 2 0 0 2 1 0 1 1 2 0 0 1 3 ? ? 1 3 ? ? ? ? ? 1 1 1 1 1 0 1 1 1 0 ? ? 1 0 ? ? 2 1 2 0 1 1 ? ? ? ? ? ? ? ? ? ? ? ? 1 1 2 0 1 1 1 1 2 0 1 0

ROM R8349

1 0 1 1 1 1 1 0 1 0 1 0 1 0 1 1 1 0 2 1 2 0 1 1 3 ? 1 1 3 ? ? ? 1 1 1 1 1 1 1 0 1 0 1 0 2 0 1&2 0 2 0 1 1 2 0 2 0 2 0 1 0 1 0 ? ? 1 1 1 1 1 0 1 1 1 1 1 1 2 0 1 0

ROM R8352

1 0 1 1 1 1 1 0 1 0 1 0 1 0 0 1 1 0 1 1 1 0 1 1 3 ? 0 1 3 ? 1 1 2 1 1 1 1 1 1 0 1 0 1 0 1 0 1 0 1 0 1 1 ? ? 1 1 ? ? ? ? ? ? ? ? ? ? ? ? 1 1 1 0 1 1 1 1 2 0 1 0

ROM R8335

1 0 0 1 1 1 1 0 1 1 1 0 2 0 0 1 1 1 1 1 1 0 1 1 3 ? 1 1 ? ? 1 1 1 1 1 1 1 1 1 1 1 1 1 0 1 0 1&2 0 1 1 1 0 ? ? ? ? 2 0 1 0 1 0 0 0 1 1 1 1 1 1 1 1 1 1 2 2 1 2 1 0

ROM R4418

0 0 0 2 0 1 0 1 0 1 1 0 1 0 1 1 0 1 0 1 2 0 1 1 3 ? 1 1 3 ? 1 1 1 1 2 1 0 1 0 1 1 1 0 0 2 1 1 0 1 1 1 1 ? ? ? ? ? ? ? ? ? ? ? ? ? ? ? ? 1 ? 1 1 2 1 1 1 1 ? 1 0

ROM R4420

0 0 0 1 0 1 1 1 0 1 0 1 0 1 0 1 1 1 0 1 1 0 0 1 3 ? 0 1 3 ? 0 1 ? ? ? ? 0 1 0 1 0 1 1 0 0 1 1 1 2 1 1 0 1 1 1 1 1 1 ? ? 1 1 0 0 1 0 1 0 1 1 ? ? 2 1 1 2 1 2 1 0

ROM R4421

1 0 1 1 0 1 1 1 0 1 0 1 0 1 0 1 1 1 1 1 0 0 0 1 3 ? 0 1 3 ? 1 1 1 1 0 1 0 1 0 1 0 1 1 0 0 1 1 1 0 1 0 1 2 1 1 1 0 1 1 0 2 1 0 0 1 0 1 0 1 1 ? ? 2 1 0 2 0 2 1 0

ROM R8331

0 0 0 1 0 1 0 0 0 1 0 1 0 1 0 1 1 1 1&2 1 0 1 0 1 3 ? 1 1 ? ? 1 1 1 1 0 1 0 2 0 1 1 1 1 0 ? ? 1 1 2 1 1 1 ? ? ? ? 2 1 2 1 1 1 0 0 1 1 1 0 1 1 2 1 2 1 1 1 0 2 1 0

ROM R4422

1 2 1 1 1 1 3 ? 1 1 1 1 1&2 1 1 1 1 0 2 1 2&3 1 1 1 3 ? 1 1 3 ? 1 1 ? ? 1 1 1 1 1 1 1 1 1 1 1 1 2 1 ? ? 1 1 1 1 1 2 2 1 1 0 1 0 0 0 1 0 1 0 1 1 2 1 2 1 1 2 1 2 1 0

ROM R1698

1 0 1 1 1 0 2 0 1 0 1 0 2 1 1 1 1 1 1 0 2 0 1 1 3 ? 2 1 3 ? 2 1 1 1 1 1 1 1 0 1 1 1 1 1 1 1 2 1 1 1 1 0 ? ? ? ? ? ? ? ? ? ? 0 0 ? ? ? ? 2 1 1 1 1 1 1 1 1 2 2 0

ROM R4405

0 0 1 1 1 1 1 0 1 0 1 0 0 0 1 1 0 0 0 1 1 0 0 1 ? ? ? ? ? ? ? ? ? ? ? ? 0 1 0 1 1 1 0 0 ? ? 1 0 ? ? 0 0 ? ? ? ? ? ? 0 0 0 0 0 0 0 1 1 0 1 1 1 1 1 1 1 1 1 2 1 0

ROM R4416

0 1 0 1 0 1 0 1 0 1 0 1 0 1 0 1 0 1 0 1 0 1 0 1 3 ? 0 1 3 ? 0 1 1 1 0 1 0 1 0 1 0 1 0 0 ? ? 1 1 ? ? 0 0 ? ? ? ? 1 1 0 0 1 1 0 0 0 1 1 0 1 1 1 1 1 1 1 2 0 2 0 0

ROM R8326

0 1 0 2 0 1 0 1 0 1 0 1 0 1 0 1 0 1 0 1 0 1 0 1 3 ? 1 1 3 ? 1 1 1 1 0 1 0 2 0 2 0 1 0 1 1 1 2 1 2 1 2 0 ? ? ? ? ? ? ? ? ? ? ? ? ? ? ? ? 1 1 1 1 1 2 1 2 0 2 0 0

ROM R4401

0 1 0 2 0 1 0 1 0 1 0 1 0 1 0 2 0 0 0 1 0 1 0 1 3 ? 1 1 3 ? 1 1 1 1 0 1 0 2 0 2 0 1 0 0 1 1 2 1 0&1 1 0 1 0 1 0 2 0 0 0 1 1 1 0 0 0 1 0 1 0 1 1 1 1 1 0 2 0 2 0 0

ROM R6253

1 0 2 0 ? ? ? ? ? ? ? ? ? ? ? ? 1 0 2 0 ? ? ? ? ? ? ? ? 3 ? 2 0 ? ? ? ? 2 0 2 0 1 0 1 0 2 0 2 0 2 0 1 0 ? ? ? ? ? ? ? ? ? ? ? ? ? ? ? ? 2 0 2 0 2 0 1 0 2 0 2 0

ROM R8355

1 1 0 1 1 0 2 0 2 1 2 0 2 0 1 1 ? 0 2 1 2 0 2 0 3 ? ? ? ? ? ? ? 2 1 2 1 1 1 1 1 1 1 1 0 ? ? 2 1 ? ? 0 0 ? ? ? ? ? ? ? ? ? ? ? ? ? ? ? ? 1 1 1 1 1 1 1 1 2 2 1 0

ROM R8332

0 0 0 1 0 1 0 0 1 0 1 0 1 0 0 1 1 1 0 1 1 1 0 1 ? ? 2 1 ? ? 2 1 2 1 1 1 0 1 0 1 1 1 1 1 ? ? 2 1 ? ? 1 0 ? ? ? ? 2 0 2 0 1 0 0 0 1 0 1 0 1 1 1 1 2 0 1 1 2 2 0 0

ROM R8347

1 1 1 1 1 1 2 0 1 1 2 0 2 0 1 0 1 1 1 1 1 1 2 1 3 ? 1 1 3 ? 1 1 1 1 1 1 1 1 1 1 1 1 2 0 0 1 2 1 1 1 1 0 ? ? ? ? ? ? ? ? ? ? ? ? ? ? ? ? 1 1 1 1 2 0 1 1 2 2 1 0

ROM R8336

0 1 0 1 0 1 0 0 0 1 0 1 0 0 0 1 1 1 1 1 0 0 0 1 2&3 ? 1 1 3 ? 1 1 1 2 0 1 0 1 0 1 1 1 1 1 1 1 1 1 1 1 2 0 ? ? ? ? 1 1 2 0 1 1 0 0 1 1 2 0 1 1 1 1 2 0 1 2 2 2 1 0

ROM R4415

0 1 0 1 0 2 0 1 0 2 0 1 0&1 1 0 1 0 1 0 2 0 1 0 1 3 ? 1 1 3 ? 1 2 1 1 0 1 0 2 0 2 2 1 0 1 0 1 0 1 1 1 0 0 ? ? ? ? ? ? ? ? ? ? ? ? ? ? ? ? 1 1 1 1 1 1 0 2 0 2 0 1

ROM R7964

? ? 1&2 0 2 0 2 0 2 0 2 0 2 0 2 0 2 0 1 0 1 0 2 0 ? ? 2 0 3 ? 1 0 ? ? 2 0 2 0 2 0 2 0 2 0 2 0 1 0 1 0 0 0 ? ? ? ? ? ? ? ? ? ? ? ? ? ? ? ? 1 0 1 0 2 0 2 0 1 0 1&2 0

ROM R7965

1 0 2 0 2 0 2 0 2 0 2 0 2 0 2 0 2 0 2 0 2 0 2 0 ? ? ? ? ? ? 2 0 ? ? ? ? 2 0 ? ? 2 0 2 0 2 0 2 0 ? ? 1 0 ? ? ? ? ? ? ? ? ? ? ? ? ? ? ? ? 2 0 2 1 1 0 2 0 0 0 2 0

ROM R7966

1 0 2 0 2 0 2 0 2 0 2 0 ? ? ? ? 2 0 2 0 ? ? ? ? ? ? ? ? ? ? ? ? ? ? ? ? 1 0 1 0 1 0 2 0 2 0 1 0 2 0 1 0 ? ? ? ? ? ? ? ? ? ? ? ? ? ? ? ? 2 0 1 0 2 0 2 0 1 0 2 0

ROM R6252

1 0 2 0 2 0 2 0 2 0 1 0 2 0 2 0 2 0 2 0 2 0 2 0 3 ? 1 0 3 ? 2 0 1 0 2 0 2 0 1 0 2 0 2 0 2 0 2 0 1 0 0 0 ? ? ? ? ? ? ? ? ? ? ? ? ? ? ? ? 1 0 2 0 2 0 2 0 2 0 2 0

ROM R8328

? ? ? ? ? ? ? ? ? ? ? ? ? ? ? ? ? ? ? ? ? ? ? ? ? ? 2 1 ? ? ? ? ? ? ? ? ? ? ? ? ? ? ? ? ? ? 1 1 1 1 2 1 ? ? ? ? ? ? ? ? ? ? ? ? ? ? ? ? 1 1 1 1 1 1 2 1 2 2 ? ?

ROM R8337

? ? ? ? ? ? ? ? ? ? ? ? ? ? ? ? ? ? ? ? ? ? ? ? ? ? 0 1 ? ? 0 1 0 1 0 2 ? ? ? ? ? ? ? ? 0 1 0 1 1 1 1 0&1 ? ? ? ? 1 1 0 1 1 1 0&1 0 1 1 0 1 0 1 1 2 1 1 1 1 0 2 0 0

ROM R8333

? ? ? ? ? ? ? ? ? ? ? ? ? ? ? ? ? ? ? ? ? ? ? ? ? ? ? ? ? ? 2 1 2 1 2 1 ? ? ? ? ? ? ? ? ? ? 2 1 2 1 2 0 ? ? ? ? ? ? ? ? ? ? ? ? ? ? ? ? 2 1 2 1 ? ? ? ? 2 2 2 0

ROM R8334

0 0 0 2 0 1 1 1 1 1 1 1 2 1 1 2 2 1 1 1 1 0 1 0 3 ? 1 1 ? ? 1 1 2 1 1 1 0 1 0 1 1 1 0&1 0 1 1 1 1 2 1 1 0 ? ? ? ? 0 1 1 0 2 1 0 0 2 0 2 0 1 1 1 1 1 1 1 1 2 2 0 0

ROM R8327

2&3 ? 0 1&2 0 1 0&1&2 1 0 1 0 1 0 1 0 1 0 1 0 1 0 1 0 1 3 ? 0 1 ? ? 0 1 0 1 0 2 0 2 0 2 0 2 0 1 ? ? 1 1 1 1 3 ? ? ? ? ? ? ? ? ? ? ? ? ? ? ? ? ? 0 2 2 1 2 1 1 2 0 2 0 0

ROM R51011

0&1&2 1 0 1 ? ? 0 1 0 1 0 1&2 1 1 ? ? ? ? ? ? ? ? ? ? 3 ? 0 1 3 ? 0 2 0 2 0 2 ? 1 ? ? ? ? ? ? ? ? 0&1&2 1 0 1 0 0 ? ? ? ? ? ? ? ? ? ? ? ? ? ? ? ? ? ? 2 0 ? ? 0 0 2 2 0 0

**S1 Table. Sutural Closure Scores and Averages for *D. novaehollandiae***


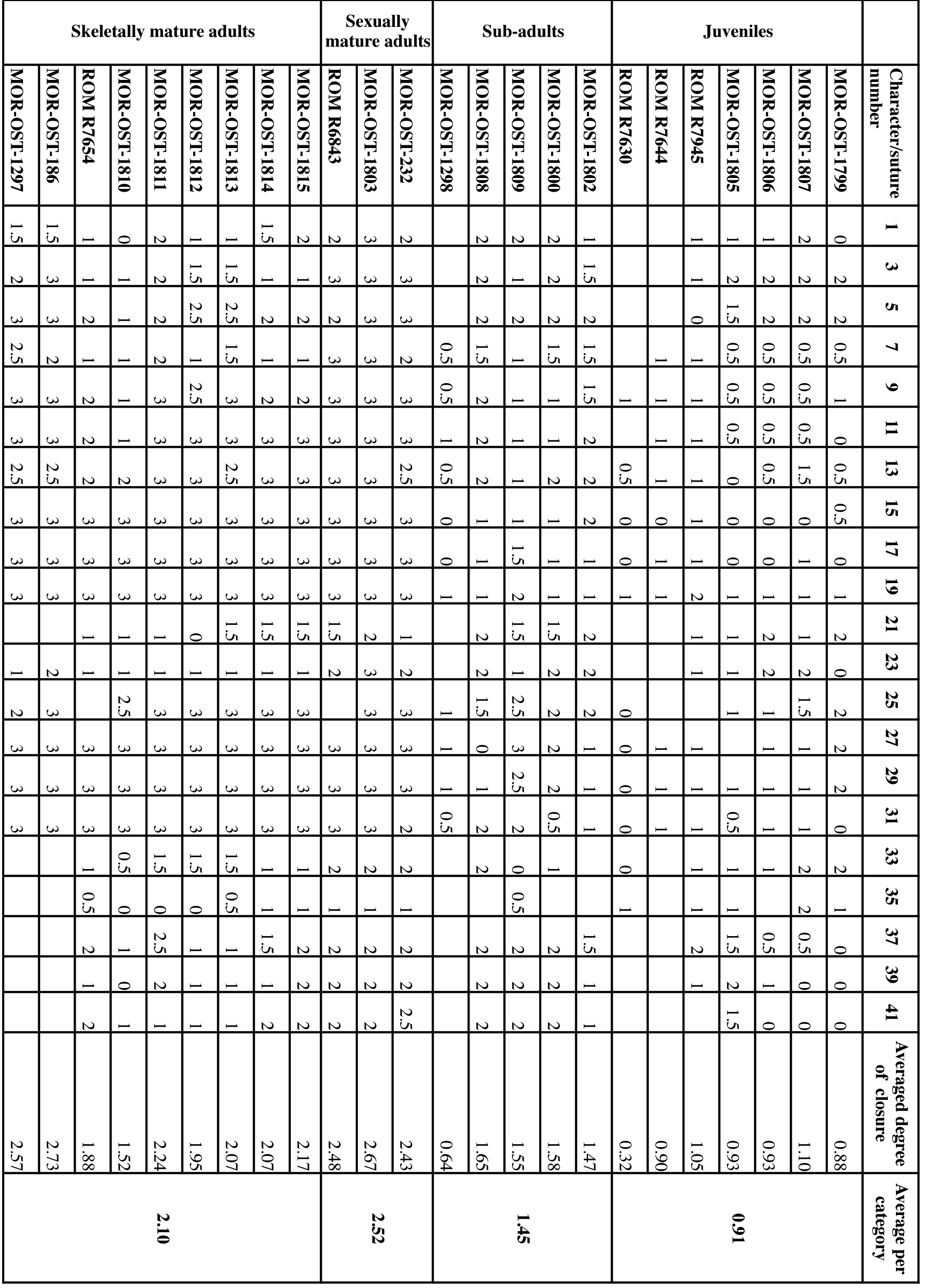


**S2 Table. Sutural Closure Scores and Averages for *A. mississippiensis***

|  | | **Character/suture number** | | | | | **1** | | **3** | | **5** | | **7** | | **9** | | **11** | | **13** | | **15** | | **17** | | **19** | | **21** | | **23** | | **25** | | **27** | | **29** | |
| --- | --- | --- | --- | --- | --- | --- | --- | --- | --- | --- | --- | --- | --- | --- | --- | --- | --- | --- | --- | --- | --- | --- | --- | --- | --- | --- | --- | --- | --- | --- | --- | --- | --- | --- | --- | --- |
| **Juveniles** | | **R6252** | | | | | 1 | | 2 | | 2 | | 2 | | 2 | | 1 | | 2 | | 2 | | 2 | | 2 | | 2 | | 2 | | 3 | | 1 | | 3 | |
|  |  | **MOR OST 1645** | | | | |  | | 1 | | 2 | | 2 | | 2 | | 2 | | 2 | | 2 | | 2 | | 1 | |  | | 1 | | 3 | | 1 | | 1.5 | |
|  |  | **R7964** | | | | |  | | 1.5 | | 2 | | 2 | | 2 | | 2 | | 2 | | 2 | | 2 | | 1 | | 1 | | 2 | |  | | 2 | | 3 | |
|  |  | **R7965** | | | | | 1 | | 2 | | 2 | | 2 | | 2 | | 2 | | 2 | | 2 | | 2 | | 2 | | 2 | | 2 | |  | |  | |  | |
|  |  | **R7966** | | | | | 1 | | 2 | | 2 | | 2 | | 2 | | 2 | |  | |  | | 2 | | 2 | |  | |  | |  | |  | |  | |
|  |  | **R6251** | | | | | 1 | | 1 | | 2 | | 1 | | 1 | | 0 | | 1 | | 0 | | 0 | | 1 | | 1 | | 2 | | 3 | | 1 | | 3 | |
|  |  | **R6253** | | | | | 1 | | 2 | |  | |  | |  | |  | |  | |  | | 1 | | 2 | |  | |  | |  | |  | | 3 | |
|  |  | **MOR-OST-148** | | | | | 2 | | 1 | | 2 | | 2 | | 1 | | 2 | | 2 | | 1 | | 1 | | 1.5 | | 2 | | 1 | | 3 | | 1 | | 3 | |
|  |  | **MOR-OST-1028** | | | | | 1 | | 1 | | 1 | | 2 | | 1 | | 1 | | 1 | | 1 | | 2 | | 1 | | 0 | | 1 | | 3 | | 0 | | 3 | |
|  |  | **MOR-OST-820** | | | | | 1 | | 0 | | 1 | | 1 | | 1 | | 1 | | 1 | | 1 | | 0 | | 0 | | 1 | | 0 | | 3 | | 0 | | 3 | |
|  |  | **R8350** | | | | | 1 | | 1 | | 1 | | 1 | | 1 | | 1 | | 2 | | 0 | | 1 | | 1 | | 2 | | 0 | | 3 | |  | | 3 | |
|  |  | **R8349** | | | | | 1 | | 1 | | 1 | | 1 | | 1 | | 1 | | 1 | | 1 | | 1 | | 2 | | 2 | | 1 | | 3 | | 1 | | 3 | |
|  |  | **R8352** | | | | | 1 | | 1 | | 1 | | 1 | | 1 | | 1 | | 1 | | 0 | | 1 | | 1 | | 1 | | 1 | | 3 | | 0 | | 3 | |
|  |  | **R8354** | | | | | 1 | | 1 | | 1 | | 1 | | 1 | | 1 | | 1 | | 1 | | 1 | | 1 | | 1 | | 1 | | 3 | | 1 | | 3 | |
|  |  | **R8355** | | | | | 1 | | 0 | | 1 | | 2 | | 2 | | 2 | | 2 | | 1 | |  | | 2 | | 2 | | 2 | | 3 | |  | |  | |
| **Sub-adults** | | **MOR-OST-1029** | | | | | 0 | | 0.5 | | 0 | | 2 | | 2 | | 1 | | 1 | | 1 | | 1 | | 1 | | 1 | | 0 | | 3 | | 1 | | 3 | |
|  |  | **R8332** | | | | | 0 | | 0 | | 0 | | 0 | | 1 | | 1 | | 1 | | 0 | | 1 | | 0 | | 1 | | 0 | |  | | 2 | |  | |
|  |  | **R8347** | | | | | 1 | | 1 | | 1 | | 2 | | 1 | | 2 | | 2 | | 1 | | 1 | | 1 | | 1 | | 2 | | 3 | | 1 | | 3 | |
|  |  | **R8322** | | | | | 1 | | 1 | | 1 | | 1 | | 1 | | 1 | | 1 | | 1 | | 1 | | 1 | | 1 | | 1 | | 3 | |  | |  | |
|  |  | **R8345** | | | | | 1 | | 1 | | 1 | | 1 | | 1 | | 1 | | 1 | | 1 | | 1 | | 1 | | 1 | | 1 | | 3 | | 1 | | 3 | |
|  |  | **R8335** | | | | | 1 | | 0 | | 1 | | 1 | | 1 | | 1 | | 2 | | 0 | | 1 | | 1 | | 1 | | 1 | | 3 | | 1 | |  | |
|  |  | **R4418** | | | | | 0 | | 0 | | 0 | | 0 | | 0 | | 1 | | 1 | | 1 | | 0 | | 0 | | 2 | | 1 | | 3 | | 1 | | 3 | |
|  |  | **R4420** | | | | | 0 | | 0 | | 0 | | 1 | | 0 | | 0 | | 0 | | 0 | | 1 | | 0 | | 1 | | 0 | | 3 | | 0 | | 3 | |
|  |  | **R1698** | | | | | 1 | | 1 | | 1 | | 2 | | 1 | | 1 | | 2 | | 1 | | 1 | | 1 | | 2 | | 1 | | 3 | | 2 | | 3 | |
|  |  | **R4405** | | | | | 0 | | 1 | | 1 | | 1 | | 1 | | 1 | | 0 | | 1 | | 0 | | 0 | | 1 | | 0 | |  | |  | |  | |
|  |  | **R8334** | | | | | 0 | | 0 | | 0 | | 1 | | 1 | | 1 | | 2 | | 1 | | 2 | | 1 | | 1 | | 1 | | 3 | | 1 | |  | |
| **Sexually mature adults** | | **MOR-OST-156** | | | | | 1 | | 0 | | 0 | | 1 | | 0.5 | | 0 | | 0 | | 0 | | 0 | | 0 | | 0 | | 0 | | 3 | | 0 | | 3 | |
|  |  | **R8323** | | | | |  | |  | |  | | 1 | | 0 | | 0 | | 0 | | 0 | | 0 | | 0 | | 0 | | 0 | | 3 | |  | |  | |
|  |  | **R8344** | | | | | 0 | | 0 | | 0 | | 1 | | 1 | | 1 | | 1 | | 1 | | 1 | | 0 | | 1 | | 1 | |  | | 1 | |  | |
|  |  | **R8342** | | | | | 0 | | 0 | | 1 | | 1 | | 0 | | 1 | | 1 | | 0 | | 1 | | 1 | | 0 | | 1 | | 3 | | 1 | | 3 | |
|  |  | **R8343** | | | | |  | | 0 | | 0 | | 0 | | 1 | | 0 | | 0 | | 0 | | 1 | | 1 | | 1 | | 0 | | 3 | | 1 | | 3 | |
|  |  | **R4421** | | | | | 1 | | 1 | | 0 | | 1 | | 0 | | 0 | | 0 | | 0 | | 1 | | 1 | | 0 | | 0 | | 3 | | 0 | | 3 | |
|  |  | **R8331** | | | | | 0 | | 0 | | 0 | | 0 | | 0 | | 0 | | 0 | | 0 | | 1 | | 1.5 | | 0 | | 0 | | 3 | | 1 | |  | |
|  |  | **R4422** | | | | | 1 | | 1 | | 1 | | 3 | | 1 | | 1 | | 1.5 | | 1 | | 1 | | 2 | | 2.5 | | 1 | | 3 | | 1 | | 3 | |
|  |  | **R4416** | | | | | 0 | | 0 | | 0 | | 0 | | 0 | | 0 | | 0 | | 0 | | 0 | | 0 | | 0 | | 0 | | 3 | | 0 | | 3 | |
|  |  | **R4401** | | | | | 0 | | 0 | | 0 | | 0 | | 0 | | 0 | | 0 | | 0 | | 0 | | 0 | | 0 | | 0 | | 3 | | 1 | | 3 | |
|  |  | **R8336** | | | | | 0 | | 0 | | 0 | | 0 | | 0 | | 0 | | 0 | | 0 | | 1 | | 1 | | 0 | | 0 | | 2.5 | | 1 | | 3 | |
| **Skeletally mature adults** | | **R8327** | | | | | 2.5 | | 0 | | 0 | | 1 | | 0 | | 0 | | 0 | | 0 | | 0 | | 0 | | 0 | | 0 | | 3 | | 0 | |  | |
|  |  | **R8326** | | | | | 0 | | 0 | | 0 | | 0 | | 0 | | 0 | | 0 | | 0 | | 0 | | 0 | | 0 | | 0 | | 3 | | 1 | | 3 | |
|  |  | **MOR-OST-155** | | | | | 0 | | 0 | | 0 | | 0 | | 1 | | 0 | | 1.5 | | 0 | | 1 | | 1 | | 0 | | 0 | | 3 | | 1 | | 3 | |
|  |  | **MOR-OST-795** | | | | |  | |  | |  | |  | | 0 | | 0 | | 0 | | 0 | | 0 | | 0 | | 0 | | 0 | | 3 | | 0 | | 3 | |
|  |  | **R4415** | | | | | 0 | | 0 | | 0 | | 0 | | 0 | | 0 | | 0.5 | | 0 | | 0 | | 0 | | 0 | | 0 | | 3 | | 1 | | 3 | |
|  |  | **R8329** | | | | | 1 | | 0 | | 0 | | 1 | | 1 | | 0 | | 0 | | 0 | | 0 | | 0 | | 1 | | 0 | | 3 | | 1 | |  | |
|  |  | **R4411** | | | | | 0 | | 0 | | 0 | | 0 | | 0 | | 0 | | 0 | | 0 | | 0 | | 0 | | 0 | | 0 | | 2.5 | | 0 | | 3 | |
|  |  | **R8337** | | | | |  | |  | |  | |  | |  | |  | |  | |  | |  | |  | |  | |  | |  | | 0 | |  | |
|  |  | **R8324** | | | | | 0 | | 0 | | 0 | | 0 | | 0 | | 0 | | 0 | | 0 | | 0 | | 0 | | 0 | | 0 | | 3 | | 0 | | 3 | |
| **31** | **33** | | **35** | **37** | **39** | **41** | | **43** | | **45** | | **47** | | **49** | | **51** | | **53** | | **55** | | **57** | | **59** | | **61** | | **63** | | **65** | | **67** | | **69** | |  |
| 2 | 1 | | 2 | 2 | 1 | 2 | | 2 | | 2 | | 2 | | 1 | | 0 | |  | |  | |  | |  | |  | |  | |  | |  | | 1 | |  |
| 1 | 1 | |  | 2 | 1 | 1 | | 2 | | 1 | | 1 | | 0.5 | | 1 | |  | |  | |  | |  | |  | |  | |  | |  | | 0 | |  |
| 1 |  | | 2 | 2 | 2 | 2 | | 2 | | 2 | | 1 | | 1 | | 0 | |  | |  | |  | |  | |  | |  | |  | |  | | 1 | |  |
| 2 |  | |  | 2 |  | 2 | | 2 | | 2 | | 2 | |  | | 1 | |  | |  | |  | |  | |  | |  | |  | |  | | 2 | |  |
|  |  | |  | 1 | 1 | 1 | | 2 | | 2 | | 1 | | 2 | | 1 | |  | |  | |  | |  | |  | |  | |  | |  | | 2 | |  |
| 2 | 2 | | 1 | 2 | 1 | 2 | | 1 | | 1 | | 2 | | 1 | | 2 | |  | |  | |  | |  | |  | |  | |  | |  | | 1 | |  |
| 2 |  | |  | 2 | 2 | 1 | | 1 | | 2 | | 2 | | 2 | | 1 | |  | |  | |  | |  | |  | |  | |  | |  | | 2 | |  |
| 2 | 2 | | 1 | 2 | 2 | 1 | | 1 | | 2 | | 2 | | 2 | | 1 | |  | |  | |  | |  | |  | |  | |  | |  | | 2 | |  |
| 1 | 2 | | 0 | 2 | 1 | 1 | | 2 | | 1 | | 2 | | 2 | | 1 | |  | |  | |  | |  | |  | |  | |  | |  | | 2 | |  |
| 1 | 2 | | 0 | 0 | 1 | 0 | | 1 | | 2 | | 2 | | 2 | | 1 | |  | |  | |  | |  | |  | |  | |  | |  | | 0 | |  |
|  |  | | 1 | 1 | 1 | 1 | | 1 | |  | | 1 | |  | | 2 | | 2 | | 1 | |  | |  | |  | |  | |  | |  | | 1 | |  |
|  | 1 | | 1 | 1 | 1 | 1 | | 1 | | 2 | | 1.5 | | 2 | | 1 | | 2 | | 2 | | 2 | | 1 | | 1 | |  | | 1 | | 1 | | 1 | |  |
| 1 | 2 | | 1 | 1 | 1 | 1 | | 1 | | 1 | | 1 | | 1 | | 1 | |  | | 1 | |  | |  | |  | |  | |  | |  | | 1 | |  |
| 1 | 1 | | 1 | 1 | 1 | 1 | | 1 | | 1 | | 1 | |  | | 1 | |  | |  | |  | |  | |  | |  | |  | |  | | 2 | |  |
|  | 2 | | 2 | 1 | 1 | 1 | | 1 | |  | | 2 | |  | | 0 | |  | |  | |  | |  | |  | |  | |  | |  | | 1 | |  |
| 1 | 0 | | 0 | 0 | 1 | 1 | | 1 | | 1 | | 2 | | 1 | | 2 | |  | |  | |  | |  | |  | |  | |  | |  | | 1 | |  |
| 2 | 2 | | 1 | 0 | 0 | 1 | | 1 | |  | | 2 | |  | | 1 | |  | |  | | 2 | | 2 | | 1 | | 0 | | 1 | | 1 | | 1 | |  |
| 1 | 1 | | 1 | 1 | 1 | 1 | | 2 | | 0 | | 2 | | 1 | | 1 | |  | |  | |  | |  | |  | |  | |  | |  | | 1 | |  |
|  |  | | 1 | 1 | 1 | 1 | | 1 | | 0 | |  | |  | | 1 | |  | |  | | 1 | | 1 | | 2 | | 0 | | 1 | | 1 | | 1 | |  |
| 1 | 1 | | 1 | 1 | 1 | 1 | | 1 | | 1 | | 2 | |  | | 1 | |  | |  | |  | |  | |  | |  | |  | |  | | 1 | |  |
| 1 | 1 | | 1 | 1 | 1 | 1 | | 1 | | 1 | | 1.5 | | 1 | | 1 | |  | |  | | 2 | | 1 | | 1 | | 0 | | 1 | | 1 | | 1 | |  |
| 1 | 1 | | 2 | 0 | 0 | 1 | | 0 | | 2 | | 1 | | 1 | | 1 | |  | |  | |  | |  | |  | |  | |  | |  | | 1 | |  |
| 0 |  | |  | 0 | 0 | 0 | | 1 | | 0 | | 1 | | 2 | | 1 | | 1 | | 1 | | 1 | |  | | 1 | | 0 | | 1 | | 1 | | 1 | |  |
| 2 | 1 | | 1 | 1 | 0 | 1 | | 1 | | 1 | | 2 | | 1 | | 1 | |  | |  | |  | |  | |  | | 0 | |  | |  | | 2 | |  |
|  |  | |  | 0 | 0 | 1 | | 0 | |  | | 1 | |  | | 0 | |  | |  | |  | | 0 | | 0 | | 0 | | 0 | | 1 | | 1 | |  |
| 1 | 2 | | 1 | 0 | 0 | 1 | | 0.5 | | 1 | | 1 | | 2 | | 1 | |  | |  | | 0 | | 1 | | 2 | | 0 | | 2 | | 2 | | 1 | |  |
| 0 | 0 | | 0 | 0 | 0 | 0 | | 0 | | 1 | | 0 | | 0 | | 0 | |  | |  | |  | |  | |  | |  | |  | |  | | 0 | |  |
|  | 0 | | 0 |  | 0 | 0 | | 0 | |  | |  | |  | | 1 | |  | |  | | 1 | | 0 | | 0 | | 0 | | 1 | | 0 | | 0 | |  |
| 3 | 1 | | 1 | 1 | 0 | 1 | | 0 | |  | | 1 | | 1 | | 0 | |  | |  | |  | |  | |  | |  | |  | |  | | 1 | |  |
| 3 | 0 | | 1 | 0 | 0 | 1 | | 1 | | 1 | | 1 | | 1 | |  | |  | |  | |  | |  | |  | |  | |  | |  | | 1 | |  |
|  | 1 | | 1 | 0 | 0 | 0 | | 0 | | 2 | | 1 | | 1 | | 0 | | 1 | | 1 | | 1 | | 0 | | 1 | | 0 | | 0 | | 0 | | 2 | |  |
| 1 | 1 | | 0 | 0 | 0 | 0 | | 1 | | 0 | | 1 | | 0 | | 0 | | 2 | | 1 | | 0 | | 1 | | 2 | | 0 | | 1 | | 1 | | 1 | |  |
| 1 | 1 | | 0 | 0 | 0 | 1 | | 1 | |  | | 1 | | 2 | | 1 | |  | |  | | 2 | | 2 | | 1 | | 0 | | 1 | | 1 | | 1 | |  |
| 1 |  | | 1 | 1 | 1 | 1 | | 1 | | 1 | | 2 | |  | | 1 | | 1 | | 1 | | 2 | | 1 | | 1 | | 0 | | 1 | | 1 | | 1 | |  |
| 0 | 1 | | 0 | 0 | 0 | 0 | | 0 | |  | | 1 | |  | | 0 | |  | |  | | 1 | | 0 | | 1 | | 0 | | 0 | | 1 | | 1 | |  |
| 1 | 1 | | 0 | 0 | 0 | 0 | | 0 | | 1 | | 2 | | 0.5 | | 0 | | 0 | | 0 | | 0 | | 0 | | 1 | | 0 | | 0 | | 0 | | 0 | |  |
| 1 | 1 | | 0 | 0 | 0 | 1 | | 1 | | 1 | | 1 | | 1 | | 2 | |  | |  | | 1 | | 2 | | 1 | | 0 | | 1 | | 2 | | 1 | |  |
| 0 | 0 | | 0 | 0 | 0 | 0 | | 0 | |  | | 1 | | 1 | | 3 | |  | |  | |  | |  | |  | |  | |  | |  | | 0 | |  |
| 1 | 1 | | 0 | 0 | 0 | 0 | | 0 | | 1 | | 2 | | 2 | | 2 | |  | |  | |  | |  | |  | |  | |  | |  | | 1 | |  |
| 2 | 2 | | 1 | 0 | 0 | 0 | | 0 | | 1 | | 1 | | 0 | | 1 | |  | |  | |  | |  | |  | |  | |  | |  | | 1 | |  |
| 0 | 0 | | 0 |  |  | 0 | |  | | 1 | | 0 | | 0 | | 0 | |  | |  | |  | |  | |  | |  | |  | |  | | 0 | |  |
| 1 | 1 | | 0 | 0 | 0 | 2 | | 0 | | 0 | | 0 | | 1 | | 0 | |  | |  | |  | |  | |  | |  | |  | |  | | 1 | |  |
| 0 | 0 | | 0 | 0 | 0 | 1 | | 1 | |  | | 2 | | 2 | | 1 | |  | |  | | 2 | | 1 | | 2 | | 1 | | 2 | | 2 | | 1 | |  |
| 0 | 0 | | 0 | 0 | 0 | 0 | | 0 | | 0 | | 0 | | 1 | | 1 | | 1 | | 0 | | 1 | | 0 | | 0 | | 0 | | 0 | | 0 | | 0 | |  |
| 0 | 0 | | 0 |  |  |  | |  | | 0 | | 0 | | 1 | | 1 | |  | |  | | 1 | | 0 | | 1 | | 0.5 | | 1 | | 0 | | 0 | |  |
| 0 | 0 | | 0 | 0 | 0 | 0 | | 0 | | 0 | | 0 | | 1 | | 1 | |  | |  | | 1 | | 1 | | 1 | | 0 | | 1 | | 1 | | 1 | |  |

| **71** | **73** | **75** | **77** | **79** | **Degree of closure (averaged)** | **Average per category** |
| --- | --- | --- | --- | --- | --- | --- |
| 2 | 2 | 2 | 2 | 2 | 1.78 | **1.43** |
| 0 | 1 | 0 | 1 | 1 | 1.28 |  |
| 1 | 2 | 2 | 1 | 1.5 | 1.66 |  |
| 2 | 1 | 2 | 0 | 2 | 1.80 |  |
| 1 | 2 | 2 | 1 | 2 | 1.64 |  |
| 2 | 1 | 1 | 2 | 2 | 1.38 |  |
| 2 | 2 | 1 | 2 | 2 | 1.75 |  |
| 2 | 2 | 2 | 0 | 2 | 1.67 |  |
| 1 | 2 | 2 | 2 | 2 | 1.41 |  |
| 1 | 0 | 1 | 2 | 1.5 | 0.98 |  |
| 2 | 1 | 1 | 2 | 1 | 1.28 |  |
| 1 | 1 | 1 | 2 | 1 | 1.33 |  |
| 1 | 1 | 1 | 2 | 1 | 1.12 |  |
| 2 | 2 | 1 | 2 | 1 | 1.26 |  |
| 1 | 1 | 1 | 2 | 1 | 1.42 |  |
| 1 | 1 | 1 | 0 | 1 | 1.02 | **1.02** |
| 1 | 2 | 1 | 2 | 0 | 0.91 |  |
| 1 | 2 | 1 | 2 | 1 | 1.34 |  |
| 2 | 1 | 1 | 0 | 1 | 1.03 |  |
| 2 | 2 | 1 | 2 | 1 | 1.26 |  |
| 1 | 1 | 2 | 1 | 1 | 1.07 |  |
| 1 | 2 | 1 | 1 | 1 | 0.94 |  |
|  | 2 | 1 | 1 | 1 | 0.72 |  |
| 1 | 1 | 1 | 1 | 2 | 1.30 |  |
| 1 | 1 | 1 | 1 | 1 | 0.55 |  |
| 1 | 1 | 1 | 2 | 0 | 1.04 |  |
| 0 | 1 | 0 | 0 | 0 | 0.33 | **0.69** |
| 1 | 0 | 0 | 0 | 0 | 0.29 |  |
| 1 | 1 | 1 | 0 | 0 | 0.76 |  |
| 2 | 1 | 1 | 0 | 0 | 0.90 |  |
| 1 | 2 | 1 | 0 | 0 | 0.71 |  |
|  | 2 | 0 | 0 | 1 | 0.69 |  |
| 2 | 2 | 1 | 0 | 1 | 0.79 |  |
| 2 | 2 | 1 | 1 | 1 | 1.32 |  |
| 1 | 1 | 1 | 0 | 0 | 0.42 |  |
| 1 | 1 | 0 | 0 | 0 | 0.39 |  |
| 1 | 2 | 1 | 2 | 1 | 0.86 |  |
| 2 | 2 | 1 | 0 | 0 | 0.55 | **0.52** |
| 1 | 1 | 1 | 0 | 0 | 0.63 |  |
| 2 | 2 | 0 | 0 | 0 | 0.77 |  |
| 1 | 1.5 | 0 | 0 | 0 | 0.38 |  |
| 1 | 1 | 0 | 0 | 0 | 0.48 |  |
| 1 | 2 | 0 | 0 | 0 | 0.81 |  |
| 0 | 1 | 0 | 0 | 0 | 0.26 |  |
| 1 | 1 | 1 | 0 | 0 | 0.43 |  |
| 1 | 0 | 0 | 0 | 0 | 0.39 |  |

**S3 Table. Sutural Interdigitation Scores and Averages for *D. novaehollandiae***


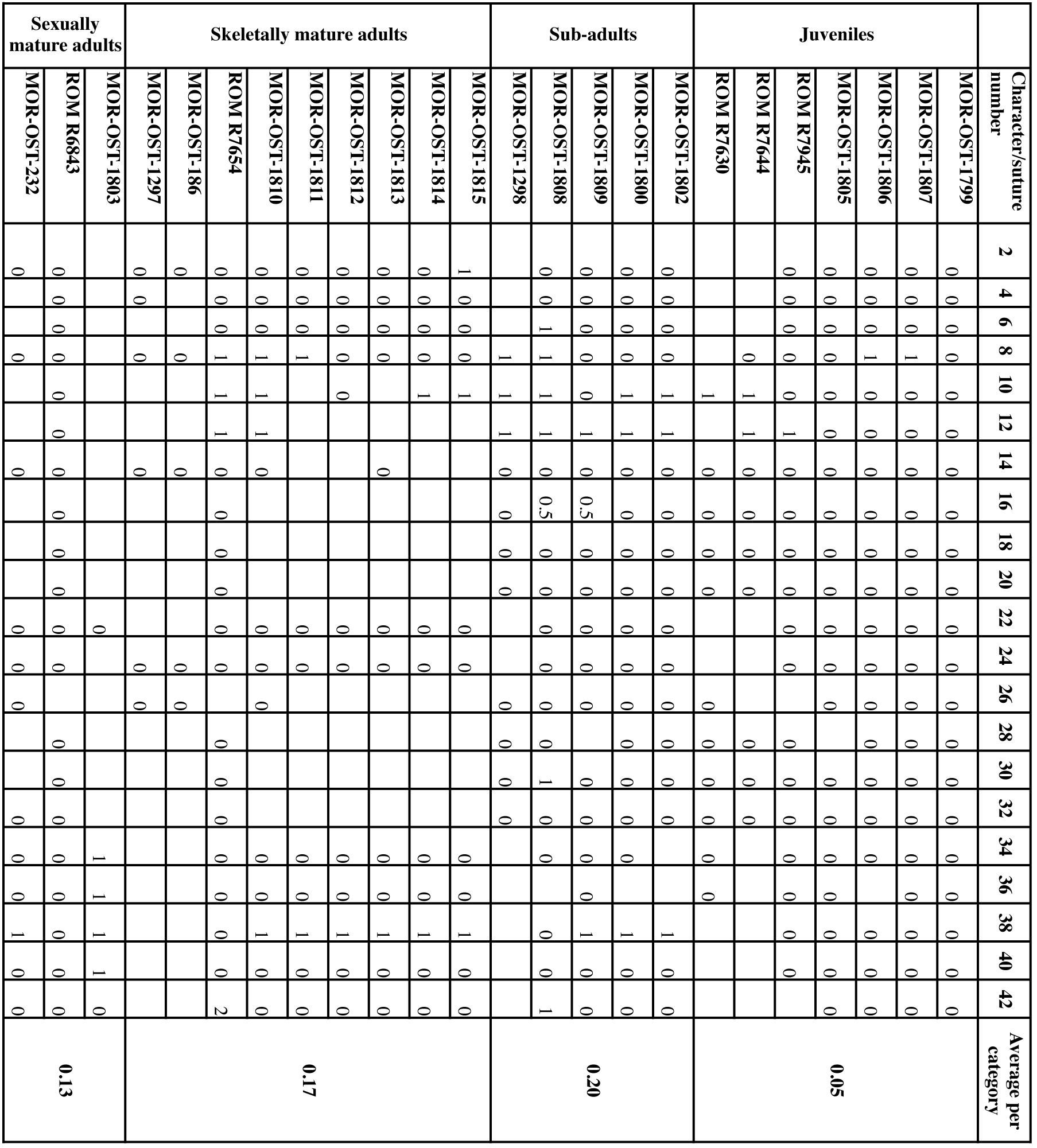


**S4 Table. Sutural Interdigitation Scores and Averages for *A. mississippiensis***

|  | **Character/suture number** | **2** | **4** | **6** | **8** | **10** | **12** | **14** | **16** | **18** | **20** | **22** | **24** | **26** | **28** | **30** | **32** | **34** |
| --- | --- | --- | --- | --- | --- | --- | --- | --- | --- | --- | --- | --- | --- | --- | --- | --- | --- | --- |
| **Juveniles** | **R6252** | 0 | 0 | 0 | 0 | 0 | 0 | 0 | 0 | 0 | 0 | 0 | 0 |  | 0 |  | 0 | 0 |
|  | **MOR OST 1645** |  | 0 | 0 | 0 | 0 | 0 | 0 | 0 | 0 | 0 | 0 | 0 |  | 0 |  | 0 | 1 |
|  | **R7964** |  | 0 | 0 | 0 | 0 | 0 | 0 | 0 | 0 | 0 | 0 | 0 |  | 0 |  | 0 |  |
|  | **R7965** | 0 | 0 | 0 | 0 | 0 | 0 | 0 | 0 | 0 | 0 | 0 | 0 |  |  |  | 0 |  |
|  | **R7966** | 0 | 0 | 0 | 0 | 0 | 0 |  |  | 0 | 0 |  |  |  |  |  |  |  |
|  | **R6251** | 0 | 1 | 0 | 0 | 0 | 1 | 0 | 0 | 0 | 0 | 1 | 0 |  | 1 |  | 0 | 1 |
|  | **R6253** | 0 | 0 |  |  |  |  |  |  | 0 | 0 |  |  |  |  |  | 0 |  |
|  | **MOR-OST-148** | 0 | 1 | 0 | 0 | 0 | 0 | 0 | 0 | 0 | 0 | 0 | 0 |  | 0 |  | 0 | 0 |
|  | **MOR-OST-1028** | 0 | 1 | 0 | 0 | 0 | 0 | 0 | 0 | 0 | 0 | 1 | 1 |  | 1 |  | 1 | 0 |
|  | **MOR-OST-820** | 0 | 1 | 0 | 0 | 0 | 0 | 0 | 1 | 0 | 1 | 1 | 1 |  | 1 |  | 0 | 0 |
|  | **R8350** | 0 | 1 | 1 | 0 | 0 | 1 | 0 | 2 | 0 | 1 | 0 | 1 |  | 1 |  |  |  |
|  | **R8349** | 0 | 1 | 1 | 0 | 0 | 0 | 0 | 1 | 0 | 1 | 0 | 1 |  | 1 |  |  | 1 |
|  | **R8352** | 0 | 1 | 1 | 0 | 0 | 0 | 0 | 1 | 0 | 1 | 0 | 1 |  | 1 |  | 1 | 1 |
|  | **R8354** | 0 | 1 | 0 | 0 | 1 | 1 | 1 | 1 | 0 | 1 | 1 | 1 |  | 1 |  | 1 | 1 |
|  | **R8355** | 1 | 1 | 0 | 0 | 1 | 0 | 0 | 1 | 0 | 1 | 0 | 0 |  |  |  |  | 1 |
| **Sub-adults** | **MOR-OST-1029** | 0 | 1 | 0.5 | 0 | 1 | 1 | 0 | 1 | 1 | 1 | 1 | 1 |  | 1 |  | 1 | 1 |
|  | **R8332** | 0 | 1 | 1 | 0 | 0 | 0 | 0 | 1 | 1 | 1 | 1 | 1 |  | 1 |  | 1 | 1 |
|  | **R8347** | 1 | 1 | 1 | 0 | 1 | 0 | 0 | 0 | 1 | 1 | 1 | 1 |  | 1 |  | 1 | 1 |
|  | **R8322** | 0 | 1 | 1 | 0 | 1 | 0 | 0 | 1 | 0 | 0 | 0 | 1 |  |  |  | 1 | 1 |
|  | **R8345** | 0 | 1 | 0 | 0 | 0 | 1 | 1 | 1 | 0 | 1 | 1 | 1 |  | 1 |  | 1 | 1 |
|  | **R8335** | 0 | 1 | 1 | 0 | 1 | 0 | 0 | 1 | 1 | 1 | 0 | 1 |  | 1 |  | 1 | 1 |
|  | **R4418** | 0 | 2 | 1 | 1 | 1 | 0 | 0 | 1 | 1 | 1 | 0 | 1 |  | 1 |  | 1 | 1 |
|  | **R4420** | 0 | 1 | 1 | 1 | 1 | 1 | 1 | 1 | 1 | 1 | 0 | 1 |  | 1 |  | 1 |  |
|  | **R1698** | 0 | 1 | 0 | 0 | 0 | 0 | 1 | 1 | 1 | 0 | 0 | 1 |  | 1 |  | 1 | 1 |
|  | **R4405** | 0 | 1 | 1 | 0 | 0 | 0 | 0 | 1 | 0 | 1 | 0 | 1 |  |  |  |  |  |
|  | **R8334** | 0 | 2 | 1 | 1 | 1 | 1 | 1 | 2 | 1 | 1 | 0 | 0 |  | 1 |  | 1 | 1 |
| **Sexually mature adults** | **MOR-OST-156** | 0 | 2 | 2 | 0 | 1 | 1 | 1 | 2 | 1 | 1 | 1 | 1 |  | 1 |  | 1 | 2 |
|  | **R8323** |  |  | 1 | 0 | 0 | 0 | 0 | 1 | 0 | 1 | 1 | 1 |  |  |  | 1 | 1 |
|  | **R8344** | 0 | 1 | 1 | 0 | 0 | 0 | 0 | 1 | 0 | 1 | 1 | 1 |  | 1 |  |  | 1 |
|  | **R8342** | 1 | 1 | 1 | 1 | 1 | 1 | 0 | 2 | 0 | 1 | 1 | 1 |  | 1 |  | 1 | 1 |
|  | **R8343** |  | 2 | 2 | 1 | 1 | 1 | 1 | 2 | 1 | 1 | 1 | 1 |  | 1 |  |  | 1 |
|  | **R4421** | 0 | 1 | 1 | 1 | 1 | 1 | 1 | 1 | 1 | 1 | 0 | 1 |  | 1 |  | 1 | 1 |
|  | **R4422** | 2 | 1 | 1 |  | 1 | 1 | 1 | 1 | 0 | 1 | 1 | 1 |  | 1 |  | 1 |  |
|  | **R4401** | 1 | 2 | 1 | 1 | 1 | 1 | 1 | 2 | 0 | 1 | 1 | 1 |  | 1 |  | 1 | 1 |
|  | **R4416** | 1 | 1 | 1 | 1 | 1 | 1 | 1 | 1 | 1 | 1 | 1 | 1 |  | 1 |  | 1 | 1 |
|  | **R8331** | 0 | 1 | 1 | 0 | 1 | 1 | 1 | 1 | 1 | 1 | 1 | 1 |  | 1 |  | 1 | 1 |
|  | **R8336** | 1 | 1 | 1 | 0 | 1 | 1 | 0 | 1 | 1 | 1 | 0 | 1 |  | 1 |  | 1 | 2 |
| **Skeletally mature adults** | **R8329** | 0 | 2 | 2 | 1 | 1 | 1 | 0 | 1 | 1 | 2 | 1 | 1 |  | 1 |  | 1 | 1 |
|  | **R4411** | 0 | 2 | 1 | 1 | 1 | 1 | 1 | 1 | 1 | 1 | 1 | 1 |  | 1 |  | 2 | 2 |
|  | **R8324** | 2 | 2 | 1 | 2 | 1 | 1 | 1 | 2 | 1 | 1 | 1 | 2 |  | 1 |  | 1 | 2 |
|  | **R8326** | 1 | 2 | 1 | 1 | 1 | 1 | 1 | 1 | 1 | 1 | 1 | 1 |  | 1 |  | 1 | 1 |
|  | **MOR-OST-155** | 0 | 2 | 2 | 1 | 1 | 1 | 0 | 1 | 1 | 1 | 1 | 1 |  | 1 |  | 1 | 2 |
|  | **MOR-OST-795** |  | 2 | 2 | 1 | 1 | 2 | 1 | 2 | 1 | 1 | 2 | 2 |  | 2 |  | 2 | 2 |
|  | **R4415** | 1 | 1 | 2 | 1 | 2 | 1 | 1 | 1 | 1 | 2 | 1 | 1 |  | 1 |  | 2 | 1 |
|  | **R8328** |  |  |  |  |  |  |  |  |  |  |  |  |  | 1 |  |  |  |
|  | **R8337** |  |  |  |  |  |  |  |  |  |  |  |  |  | 1 |  | 1 | 1 |
|  | **R8333** |  |  |  |  |  |  |  |  |  |  |  |  |  |  |  | 1 | 1 |
|  | **R8327** |  | 1.5 | 1 | 1 | 1 | 1 | 1 | 1 | 1 | 1 | 1 | 1 |  | 1 |  | 1 | 1 |

| **36** | **38** | **40** | **42** | **44** | **46** | **48** | **50** | **52** | **54** | **56** | **58** | **60** | **62** | **64** | **66** | **68** | **70** | **72** | **74** |
| --- | --- | --- | --- | --- | --- | --- | --- | --- | --- | --- | --- | --- | --- | --- | --- | --- | --- | --- | --- |
| 0 | 0 | 0 | 0 | 0 | 0 | 0 | 0 | 0 |  |  |  |  |  |  |  |  | 0 | 0 | 0 |
|  | 0 | 0 | 0 | 0 | 0 | 1 | 0 | 0 |  |  |  |  |  |  |  |  | 0 | 0 | 0 |
| 0 | 0 | 0 | 0 | 0 | 0 | 0 | 0 | 0 |  |  |  |  |  |  |  |  | 0 | 0 | 0 |
|  | 0 |  | 0 | 0 | 0 | 0 |  | 0 |  |  |  |  |  |  |  |  | 0 | 1 | 0 |
|  | 0 | 0 | 0 | 0 | 0 | 0 | 0 | 0 |  |  |  |  |  |  |  |  | 0 | 0 | 0 |
| 1 | 1 | 0 | 1 | 0 | 0 | 0 | 0 | 0 |  |  |  |  |  |  |  |  | 1 | 0 | 0 |
|  | 0 | 0 | 0 | 0 | 0 | 0 | 0 | 0 |  |  |  |  |  |  |  |  | 0 | 0 | 0 |
| 1 | 0 | 0 | 1 | 0 | 0 | 0 | 0 | 0 |  |  |  |  |  |  |  |  | 0 | 0 | 1 |
| 1 | 1 | 0 | 0 | 0 | 1 | 1 | 0 | 0 |  |  |  |  |  |  |  |  | 1 | 0 | 1 |
| 1 | 1 | 0 | 1 | 0 | 0 | 1 | 1 | 0.5 |  |  |  |  |  |  |  |  | 1 | 1 | 1 |
| 1 | 1 | 0 | 1 | 0 |  | 0 |  | 1 | 0 | 1 |  |  |  |  |  |  | 1 | 0 | 1 |
| 1 | 1 | 0 | 0 | 0 | 0 | 0 | 0 | 1 | 0 | 0 | 0 | 0 | 0 |  | 1 | 1 | 0 | 1 | 1 |
| 1 | 1 | 0 | 0 | 0 | 0 | 0 | 0 | 1 |  | 1 |  |  |  |  |  |  | 1 | 0 | 1 |
| 1 | 1 | 0 | 1 | 0 | 0 | 1 | 0 | 1 |  |  |  |  |  |  |  |  | 1 | 0 | 0 |
| 1 | 1 | 1 | 1 | 0 |  | 1 |  | 0 |  |  |  |  |  |  |  |  | 1 | 1 | 1 |
| 1 | 1 | 1 | 1 | 0 | 1 | 1 | 1 | 0 |  |  |  |  |  |  |  |  | 1 | 1 | 1 |
| 1 | 1 | 1 | 1 | 1 |  | 1 |  | 0 |  |  | 0 | 0 | 0 | 0 | 0 | 0 | 1 | 1 | 0 |
| 1 | 1 | 1 | 1 | 0 | 1 | 1 | 1 | 0 |  |  |  |  |  |  |  |  | 1 | 1 | 0 |
|  | 1 | 1 | 1 | 0 | 0 |  |  | 1 |  |  | 0 | 0 | 1 | 0 | 1 | 1 | 1 | 1 | 1 |
| 1 | 1 | 0 | 1 | 0 | 1 | 1 |  | 1 |  |  |  |  |  |  |  |  | 1 | 0 | 0 |
| 1 | 1 | 1 | 1 | 0 | 0 | 0 | 1 | 0 |  |  | 0 | 0 | 0 | 0 | 1 | 1 | 1 | 1 | 1 |
| 1 | 1 | 1 | 1 | 0 | 1 | 0 | 1 | 1 |  |  |  |  |  |  |  |  |  | 1 | 1 |
|  | 1 | 1 | 1 | 0 | 1 | 1 | 1 | 0 | 1 | 1 | 1 |  | 1 | 0 | 0 | 0 | 1 |  | 1 |
| 1 | 1 | 1 | 1 | 1 | 1 | 1 | 1 | 0 |  |  |  |  |  |  |  |  | 1 | 1 | 1 |
|  | 1 | 1 | 1 | 0 |  | 0 |  | 0 |  |  |  | 0 | 0 | 0 | 1 | 0 | 1 | 1 | 1 |
| 1 | 1 | 1 | 1 | 0 | 1 | 1 | 1 | 0 |  |  | 1 | 0 | 1 | 0 | 0 | 0 | 1 | 1 | 1 |
| 2 | 2 | 1 | 1 | 0 | 1 | 1 | 1 | 0 |  |  |  |  |  |  |  |  | 1 | 1 | 1 |
| 1 | 1 | 2 | 1 | 0 |  |  |  | 1 |  |  | 1 | 1 | 1 | 0 | 1 | 1 | 1 | 1 | 1 |
| 1 | 1 | 1 | 1 | 0 |  | 1 | 1 | 1 |  |  |  |  |  |  |  |  | 1 | 1 | 1 |
| 1 | 1 | 1 | 1 | 0 | 0 | 1 | 1 |  |  |  |  |  |  |  |  |  | 1 | 1 | 1 |
| 1 | 2 | 2 | 2 | 1 | 0 | 1 | 1 | 1 | 1 | 2 | 1 | 1 | 1 | 0 | 1 | 1 | 1 | 1 | 1 |
| 1 | 1 | 1 | 1 | 0 | 1 | 1 | 1 | 1 | 1 | 1 | 1 | 0 | 1 | 0 | 0 | 0 | 1 |  | 1 |
| 1 | 1 | 1 | 1 | 1 | 1 | 1 |  | 1 | 1 | 2 | 1 | 0 | 0 | 0 | 0 | 0 | 1 | 1 | 1 |
| 1 | 2 | 2 | 1 | 0 | 1 | 1 | 1 | 1 | 1 | 2 | 0 | 1 | 1 | 0 | 1 | 1 | 1 | 1 | 1 |
| 1 | 1 | 1 | 1 | 0 |  | 1 |  | 0 |  |  | 1 | 0 | 1 | 0 | 1 | 0 | 1 | 1 | 1 |
| 1 | 2 | 1 | 1 | 0 |  | 1 | 1 | 1 |  |  | 1 | 1 | 1 | 0 | 1 | 0 | 1 | 1 | 1 |
| 1 | 1 | 1 | 1 | 1 | 1 | 1 | 1 | 0 |  |  | 1 | 0 | 1 | 0 | 1 | 0 | 1 | 1 | 0 |
| 1 | 2 | 2 | 1 | 1 |  | 1 | 1 | 1 |  |  | 0 | 1 | 1 | 0 | 1 | 1 | 1 | 0 | 0 |
| 2 | 2 | 2 | 2 | 0 | 1 | 2 | 1 | 1 | 1 | 2 | 1 | 1 | 1 | 0 | 1 | 1 | 1 | 1 | 0 |
| 2 | 2 | 2 | 2 | 1 | 1 | 2 | 1 | 2 |  |  | 1 | 1 | 1 | 0 | 0 | 1 | 2 | 1 | 1 |
| 1 | 2 | 2 | 1 | 1 | 1 | 1 | 1 | 0 |  |  |  |  |  |  |  |  | 1 | 1 | 2 |
| 2 | 2 | 2 | 1 | 0 | 1 | 1 | 1 | 0 |  |  |  |  |  |  |  |  | 1 | 1 | 1 |
| 2 | 2 |  | 1 |  | 1 | 1 | 1 | 1 |  |  |  |  |  |  |  |  | 2 | 1 | 1 |
| 1 | 2 | 2 | 1 | 1 | 1 | 1 | 1 | 0 |  |  |  |  |  |  |  |  | 1 | 1 | 1 |
|  |  |  |  |  |  | 1 | 1 | 1 |  |  |  |  |  |  |  |  | 1 | 1 | 1 |
| 2 |  |  |  |  | 1 | 1 | 1 | 0.5 |  |  | 1 | 1 | 1 | 0 | 1 | 1 | 1 | 2 | 1 |
| 1 |  |  |  |  |  | 1 | 1 | 0 |  |  |  |  |  |  |  |  | 1 | 1 |  |
| 2 | 2 | 2 | 2 | 1 | ? | 1 | 1 |  |  |  |  |  |  |  |  |  | 2 | 1 | 1 |

| **76** | **78** | **80** | **Average per category** |
| --- | --- | --- | --- |
| 0 | 0 | 0 | **0.30** |
| 0 | 0 | 0 |  |
| 0 | 0 | 0 |  |
| 0 | 0 | 0 |  |
| 0 | 0 | 0 |  |
| 0 | 1 | 0 |  |
| 0 | 0 | 0 |  |
| 1 | 0 | 0 |  |
| 1 | 0 | 0 |  |
| 1 | 2 | 0 |  |
| 1 | 0 | 0 |  |
| 1 | 0 | 0 |  |
| 1 | 0 | 0 |  |
| 1 | 0 | 0 |  |
| 1 | 2 | 0 |  |
| 1 | 2 | 0 | **0.71** |
| 1 | 2 | 0 |  |
| 1 | 2 | 0 |  |
| 1 | 2 | 1 |  |
| 1 | 0 | 0 |  |
| 2 | 2 | 0 |  |
| 1 |  | 0 |  |
| 2 | 2 | 0 |  |
| 1 | 2 | 0 |  |
| 1 | 2 | 0 |  |
| 1 | 2 | 0 |  |
| 2 | 2 | 0 | **0.94** |
| 2 | 2 | 1 |  |
| 2 | 2 | 0 |  |
| 2 | 2 | 1 |  |
| 2 | 2 | 1 |  |
| 2 | 2 | 0 |  |
| 2 | 2 | 0 |  |
| 2 | 2 | 0 |  |
| 2 | 2 | 0 |  |
| 1 | 2 | 0 |  |
| 2 | 2 | 0 |  |
| 2 | 2 | 0 | **1.18** |
| 2 | 2 | 0 |  |
| 2 | 2 | 1 |  |
| 2 | 2 | 0 |  |
| 2 | 2 | 0 |  |
|  | 2 | 0 |  |
| 2 | 2 | 1 |  |
| 1 | 2 |  |  |
| 1 | 2 | 0 |  |
|  | 2 | 0 |  |
| 2 | 2 | 0 |  |
